# Bioinformatics insights into *TMPO-AS1–let-7b-5p–ESPL1/E2F8* regulatory axis in breast cancer

**DOI:** 10.3389/fcell.2025.1635862

**Published:** 2025-11-05

**Authors:** Rajeev Nema, Prerna Vats, Aditi Singh, Jaya Thilakan, Swagata Brahmachari, Pallavi Kulkarni, Bhavika Baweja, Chainsee Saini, Sudhir K. Goel, Neha Arya, Ashok Kumar

**Affiliations:** ^1^ Department of Biosciences, Manipal University Jaipur, Jaipur, Rajasthan, India; ^2^ Department of Biochemistry, All India Institute of Medical Sciences (AIIMS), Bhopal, Madhya Pradesh, India; ^3^ Department of General Surgery, All India Institute of Medical Sciences (AIIMS), Bhopal, Madhya Pradesh, India; ^4^ Department of Translational Medicine, All India Institute of Medical Sciences (AIIMS), Bhopal, Madhya Pradesh, India

**Keywords:** breast cancer, *ESPL1*, TCGA, metastasis, prognosis, hsa-let-7b-5, *TMPO-AS1*, ceRNA network

## Abstract

**Background:**

Breast cancer (BC) is the most frequently diagnosed malignancy in women, contributing to high morbidity and mortality rates. Dysregulation of Extra Spindle Pole Bodies Like 1 (*ESPL1*), a mitotic regulator essential for chromosomal segregation, is frequently upregulated in cancers. However, the mechanisms underlying *ESPL1* overexpression and its prognostic relevance in BC remain unclear.

**Methods:**

The study performed the data mining of The Cancer Genome Atlas (TCGA) using various web-based computational tools, including TIMER 2.0, UALCAN, FIREHOSE, TISIDB, GEPIA2, OncoDB, TCGA Portal, TCGAnalyzeR v1.0, bc-GenExMiner v5.0, TNMplot, and DriverDBv4 to compare *ESPL1* expression in tumor vs. normal tissues across pan-cancer and BC subtypes. The Kaplan-Meier (KM) Plotter database was used to determine the association between *ESPL1* expression and the survival outcomes of BC patients. miRNet, TACCO, and CancerMIRNome databases were used to analyze miRNAs correlated with *ESPL1*, while lncRNAs were analyzed using the Enrichr database. For experimental validation, *ESPL1* expression level was analyzed in BC tumor and adjacent normal tissue collected from BC patients.

**Results:**

We found that *ESPL1* gene was significantly overexpressed in tumors, metastatic tissues, and circulating tumor cells, with tumor samples showing an overall 4-fold increase in expression compared to adjacent normal tissue of BC patients. Furthermore, BC patients with high *ESPL1* expression exhibited shorter overall survival (OS), disease-free survival (DFS), and relapse-free survival (RFS) compared to patients with low expression. Tumors from ER-negative and PR-negative BC patients exhibited elevated expression levels of both *ESPL1* and the transcription factor *E2F8*. Moreover, increased levels of *ESPL1* and *E2F8* were positively correlated with lncRNA *TMPO-AS1*, while negatively correlated with *hsa-let-7b-5p*. Notably, the 3′ untranslated region (3′UTR) of *ESPL1* showed strong binding sites for *hsa-let-7b-5p*. We also identified Hesperidin as a high affinity *ESPL1* binders, suggesting novel therapeutic candidates targeting this oncogenic network.

## Graphical Abstract

**Figure:**
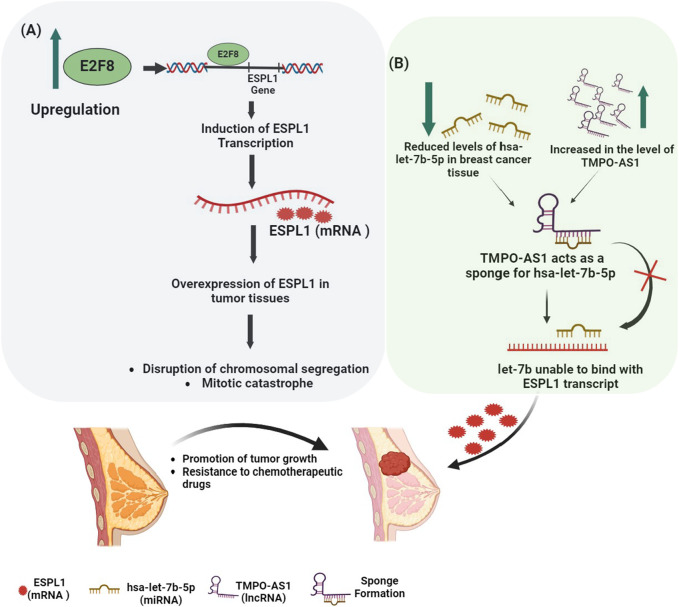
Elucidating cancer’s regulatory mechanisms behind *ESPL1* gene expression. The scheme **(A)** shows that the upregulated transcriptional factor *E2F8* causes the expression of *ESPL1* mRNA in BC when it binds to the gene’s promoter region. Overexpression of *ESPL1* messes up normal mitotic functions, resulting in chromosomal instability, mitotic catastrophe, and, finally, aggressive tumor growth. The scheme **(B)** shows how mRNA and ncRNAs are controlled, focusing on *hsa-let-7b-5p* (miRNA) and *TMPO-AS1* (lncRNA). The upregulation of *TMPO-AS1* leads to the downregulation of *hsa-let-7b-5p* by sponge formation. This prevents let-7b from binding to the miRNA regulatory element (MRE) on the *ESPL1* transcript, resulting in excessive expression of *ESPL1* in breast invasive carcinoma.

## Highlights

• *ESPL1* is overexpressed in tumors from breast cancer patients and correlates with worse survival outcomes (OS, RFS, and DMFS), highlighting its prognostic significance.
• *E2F8* transcriptional regulation of *ESPL1* reveal a cell cycle–driven axis contributing to aggressive BC subtypes.
• *hsa-let-7b-5p* downregulation in BC negatively correlates with *ESPL1*/*E2F8* levels and worse survival, supporting its role as a tumor suppressor.
• TMPO AS1 acts as a ceRNA sponge for *hsa-let-7b-5p*, relieving *ESPL1* and *E2F8* from miRNA repression and driving tumor proliferation.
• Molecular docking identifies Hesperidin as high affinity *ESPL1* binders, suggesting novel therapeutic candidates targeting this oncogenic network.

## 1 Introduction

Breast cancer (BC) is the most common type of cancer among women and is classified into four major subtypes based on histopathological and molecular biomarkers: luminal A, luminal B, HER2-positive, and triple-negative breast cancer (TNBC) (Cserni et al., 2021). Despite advances in diagnosis and treatment, the prognosis for TNBC patients remains poor due to its aggressive nature and lack of targeted therapies. Therefore, understanding the molecular mechanism of BC may help in stratifying high-risk patients and developing more effective targeted treatments. Among the hallmarks of cancer, cell cycle dysregulation plays a pivotal role, particularly during mitosis where accurate chromosome segregation is vital for genomic stability (Pati, 2024). In this context, Extra Spindle Pole Bodies-Like 1 (*ESPL1*), a cysteine endopeptidase, facilitates the separation of sister chromatids during anaphase by cleaving cohesin complexes (Cipressa et al., 2025). Notably, overexpression of *ESPL1* has been linked to chromosomal instability and aneuploidy, which are both features associated with poor prognosis in BC patients (Finetti et al., 2014). However, the mechanisms driving *ESPL1* dysregulation in BC remain incompletely understood. Thus, understanding the molecular pathways controlled by *ESPL1* is crucial for identifying novel biomarkers and therapeutic targets.

Given the heterogenous nature of breast cancer, there is an urgent need for the identification of novel molecular prognostic markers. Non-coding RNAs (ncRNAs) such as long non-coding RNAs (lncRNAs), microRNAs (miRNAs), circular RNAs (circRNAs), and PIWI-interacting RNAs (piRNAs) have been implicated in the regulation of tumorigenesis across various human cancers, including BC (Hosseinalizadeh et al., 2022). Among these, lncRNAs have gained considerable attention for their ability to act as competing endogenous RNAs (ceRNAs), sponging miRNAs to modulate gene expression, thereby influencing cancer cell proliferation, growth, migration, and invasion in cancer cells (Wu et al., 2024). One such lncRNA, TMPO antisense RNA 1 (*TMPO-AS1*), has been found to facilitate BC progression by modulating the expression of its sense transcript Thymopoietin (*TMPO*) (Wu et al., 2024). The *TMPO* gene encodes a nuclear structural protein, also known as lamina-associated polypeptide 2 (LAP2), which plays a crucial role in nuclear envelope organization, chromatin structure maintenance, and cell cycle regulation (Lunin et al., 2022). Dysregulation of *TMPO* expression has been associated with abnormal cell proliferation and cancer development (Sun et al., 2019; Huerta-Padilla et al., 2025). Importantly, *TMPO-AS1* regulates *TMPO* expression by functioning as a ceRNA, and an imbalance between *TMPO* and *TMPO-AS1* can disrupt normal gene regulation, thereby contributing to cancer progression (Li Z. et al., 2020). Furthermore, the ceRNA network, comprising mRNA, miRNA, and lncRNA interactions through shared microRNA response elements has been implicated in numerous oncogenic processes and is being investigated as a potential source of diagnostic and prognostic markers. Additionally, transcription factors from the E2F family are known to regulate cell cycle progression, proliferation, and apoptosis, and have been shown to influence BC development by controlling the expression of numerous target genes (Kassab et al., 2023).

In this study, we investigate the prognostic relevance and regulatory network of *ESPL1* in BC. Specifically, we analyzed *ESPL1*’s interactions with transcription factors, miRNAs, and lncRNAs (ceRNA network) using multiple publicly available datasets. Furthermore, to validate our computational observations, we performed qRT-PCR analysis on tumor and adjacent normal tissues obtained from BC patients. In addition, molecular docking studies were carried out to assess the binding affinities of natural and chemotherapeutic compounds with *ESPL1*, thereby exploring its potential as a therapeutic target.

## 2 Materials and methods

### 2.1 Computational analysis

#### 2.1.1 Expression analysis of *ESPL1*

To investigate the differential expression of *ESPL1* across a wide range of cancer types, we performed a comprehensive In-silico data mining analysis utilizing various publicly available resources. Expression comparisons between tumor and normal tissues were carried out using web-based platforms that integrate The Cancer Genome Atlas (TCGA) and Genotype Tissue Expression (GTEx) data. We first utilized TIMER2.0 (http://timer.cistrome.org/) (Li T. et al., 2020), UALCAN (https://ualcan.path.uab.edu) (Chandrashekar et al., 2022) and Broad Firehose GDAC (https://gdac.broadinstitute.org) (Feng et al., 2021), which provided expression profiles across ∼33 cancer types. To refine these findings within the breast cancer (BC) context, we employed UALCAN, which enables stratification by molecular subtypes (Luminal A/B, HER2-enriched, TNBC), hormone receptor status, and tumor stages. mRNA expression results were further validated using ENCORI (https://rnasysu.com/encori/) (Li et al., 2014), GEPIA2 (http://gepia2.cancer-pku.cn/#index) (Tang et al., 2019), OncoDB (https://oncodb.org) (Tang et al., 2022), and the TCGA Portal (Xu et al., 2019), which offer integrative visualizations of TCGA/GTEx-derived expression patterns in breast tumors. In parallel, differential expression analysis in R was performed using HTSeq-count files (TCGA-BRCA), normalized via the DESeq2 package (v1.38.3). Standard preprocessing steps including log2 transformation and variance stabilization were applied. A box plot generated using ggplot2 confirmed significant *ESPL1* upregulation in tumors (*n* = 114) vs. normal tissues (*n* = 114), with an adjusted FDR p-value <0.05 considered significant. For transcript-level visualization in single-cell BC conditions, we used TCGAnalyzer v1.0 (http://tcganalyzer.mu.edu.tr) (Zengin et al., 2024) while CancerSEA (http://biocc.hrbmu.edu.cn/CancerSEA/) (Yuan et al., 2019) was employed to examine *ESPL1*’s association with functional phenotypes such as proliferation and to compare it with known housekeeping genes using single-cell RNA-seq data. To assess subtype-specific expression and clinical relevance, BC-GenExMiner v5.0 (http://bcgenex.ico.unicancer.fr) (Jézéquel et al., 2021) was utilized, enabling subgroup-based analysis across ER/PR status, TNBC, and histological grade. Additionally, TNMplot (https://tnmplot.com/analysis/) (Bartha and Győrffy, 2021) facilitated evaluation of *ESPL1* expression across normal, primary, and metastatic samples using harmonized TCGA, GTEx, and TARGET datasets. Finally, DriverDBv4 (https://driverdb.tms.cmu.edu.tw/) (Liu et al., 2024) was used to explore *ESPL1* expression in the context of driver mutations and transcriptomic alterations in BC.

#### 2.1.2 Association of *ESPL1* and its co-expressed genes with the survival outcome

To evaluate the prognostic significance of *ESPL1* expression in breast cancer (BC), we utilized the Kaplan–Meier (KM) Plotter database (https://kmplot.com/analysis/index.php?p=background) (Győrffy, 2023). The analysis was performed using the probe ID 38158_at, with the following parameters: Cancer type: Breast Cancer (BC) and Gene symbol: *ESPL1*. Patients were stratified into high and low expression groups based on the median *ESPL1* expression value. Associations between *ESPL1* expression and relapse-free survival (RFS), overall survival (OS), and distant metastasis-free survival (DMFS) were assessed using Kaplan–Meier survival curves.

To identify genes co-expressed with *ESPL1*, we employed multiple integrative bioinformatics platforms including Enrichr (https://maayanlab.cloud/Enrichr/) (Xie et al., 2021), TIMER (Li et al., 2017), and GSCA (Gene Set Cancer Analysis) (https://guolab.wchscu.cn/GSCA/#/) (Liu et al., 2023). Functional enrichment analysis of *ESPL1* co-expressed genes was conducted using Enrichr, with a focus on identifying transcription factors involved in tissue-specific gene regulatory networks. Further, we explored the regulatory relationship between *ESPL1* and the E2F transcription factor family, given their known role in cell cycle regulation. Correlation analyses were performed using the ENCORI and TIMER databases. Expression patterns of individual E2F members in breast cancer tissues were profiled using the TCGAnalyzeR platform.

#### 2.1.3 Analysis of non-coding RNA associated regulatory networks

To explore the non-coding RNA regulatory mechanisms associated with *ESPL1* in breast cancer (BC), we first identified miRNAs potentially targeting *ESPL1* using the miRNet database (https://www.mirnet.ca/) (Chang and Xia, 2023). Subsequently, the predicted interactions were validated by assessing expression correlations between *ESPL1* and candidate miRNAs using ENCORI, TACCO (http://tacco.life.nctu.edu.tw/) (Chou et al., 2019), and CancerMIRNome (http://bioinfo.jialab-ucr.org/CancerMIRNome/) (Li et al., 2022). To gain further insights into their clinical relevance, we evaluated the prognostic significance of *ESPL1*-associated miRNAs using KM Plotter, ENCORI, and CancerMIRNome. In parallel, we analyzed the differential expression of these miRNAs in tumor tissues of BC patients by stratifying the data according to clinicopathological variables, such as stage, race, gender, lymph node metastasis, and molecular subtype, using CancerMIRNome, UALCAN, and ExplORRnet (https://mirna.cs.ut.ee/) (Lawarde et al., 2024). To delve deeper, we examined miRNAs potential direct interaction with *ESPL1* and the transcription factor *E2F8*. For this purpose, miRWalk (http://mirwalk.umm.uni-heidelberg.de/) (Dweep et al., 2014) and RNA22v2 (https://cm.jefferson.edu/rna22/Interactive/) (Loher and Rigoutsos, 2012) were used to predict binding affinity and secondary structure, thereby evaluating the stability and regulatory impact of these interactions. In addition to miRNAs, we sought to identify lncRNAs associated with *ESPL1*, which could potentially form a ceRNA regulatory axis. To this end, we used Enrichr and UALCAN for initial screening. The identified lncRNAs were further validated via ENCORI and miRNet, establishing putative lncRNA–miRNA–mRNA interactions. Additionally, Cytoscape software (3.10.3) (Shannon et al., 2003) was used to visualize the predicted lncRNA–miRNA–mRNA-TF regulatory network, an interaction table containing TMPO-AS1, hsa-let-7b-5p, E2F8, and ESPL1 was imported into Cytoscape. The network was configured as a directed graph to represent the regulatory flow, with node shapes and colors customized to distinguish molecular types and edges styled with directional arrows. Further, to understand the broader implications, we analyzed the pan-cancer relevance and clinicopathological associations of these lncRNAs using lncRNADisease v3.0 (http://www.rnanut.net/lncrnadisease/index.php/home/search) (Lin et al., 2024), GEPIA, UALCAN, and ENCORI. These analyses provided insights into their differential expression patterns across stages and molecular subtypes. To contextualize these findings at the transcriptomic level, we performed comprehensive expression and co-expression analyses using TCGAnalyzeR. Furthermore, we explored the relationship between the ceRNA network components, including *ESPL1*, *E2F8*, *hsa-let-7b-5p*, and *TMPO-AS1* and the hormone receptor genes *ESR1* and *PGR* using ENCORI and OncoDB. Interestingly, the analysis also revealed a strong association between *TMPO-AS1* and the proliferation marker *MKI67*, highlighting its potential role in tumor progression.

### 2.2 Experimental validation

#### 2.2.1 Subjects and tissue collection

Breast tumor and adjacent normal tissue samples were obtained from the confirmed cases of breast cancer patients (*N* = 8) enrolled at the General Surgery Department, AIIMS Bhopal TNM Staging and grade of the patients is mentioned in Supplementary Table S1. Informed written consent was obtained from all participants. Patients aged above 18 years and not subjected to chemotherapy or radiotherapy prior to surgery were included in the study. Paired tumor and adjacent normal tissues (collected ≥2 cm away from the tumor margin) were collected directly from the operation theatre at the time of surgical resection and immediately preserved in 500 μL RNAlater, followed by storage at −20 °C until RNA isolation. Ethical approval for the study was granted by the Institutional Human Ethics Committee, AIIMS Bhopal (Approval No: LOP IHEC-LOP/2017/EF0057).

#### 2.2.2 Gene expression analysis

Qualitative real-time PCR (qPCR) was used to determine the mRNA expression of *ESPL1* gene in the tumor and adjacent healthy tissue of the breast cancer patients. Total RNA was extracted using the Aurum™ Total RNA Mini Kit (Bio-Rad) following the manufacturer’s protocol. Complementary DNA was prepared from total RNA using cDNA synthesis kit (iScript, Bio-Rad Laboratories, Inc., Hercules, CA, United States of America) and qPCR was carried out using SYBR green PCR master mix (Bio-Rad Laboratories) on a Real-Time PCR System. The cycling conditions for qPCR included initial denaturation at 95 °C for 3 min, 45 cycles of denaturation at 95 °C and annealing/extension at 54 °C for 30 s. Gene expression values for *ESPL1* were normalized with respect to β Actin, and fold change was determined using 2^−ΔΔCt^ method method. Primer sequences for ESPL1 were obtained from a previously published study (Liu et al., 2021).

Following set of primers were used for the study:

β Actin Forward: 5′-GACGACATGGAGAAAATCTG-3’

β Actin Reverse: 5′-ATGATCTGGGTCATCTTCTC-3’

*ESPL1* Forward: 5′-GCCCTAAAACTTACAACAAA-3’

*ESPL1*-Reverse: 5′- AGACTCAAGCAAGAACAGAA-3’

### 2.3 Molecular docking

The crystal structure of the *ESPL1* protein was obtained from the Protein Data Bank (https://www.rcsb.org/) using PDB ID: 7NJ1. In parallel, the chemical structures of four ligands, namely, Hesperidin (CID: 10621), Quercetin (CID: 5280343), Paclitaxel (CID: 36314), and Docetaxel (CID: 148124) were retrieved from the PubChem database (https://pubchem.ncbi.nlm.nih.gov/). Prior to docking, ligand structure minimization and protein structure preparation (including removal of heteroatoms and water molecules) were carried out using UCSF Chimera (https://www.cgl.ucsf.edu/chimera/). The AutoDock Tools 1.5.7 suite (https://ccsb.scripps.edu/mgltools/downloads/; https://autodock.scripps.edu/download-autodock4/) was then employed to evaluate the molecular binding interactions between *ESPL1* and the selected ligands, which included both natural compounds and standard chemotherapeutic agents. For each ligand, ten docking conformations were generated. Among these, the top-scoring conformation, based on lowest binding energy, was selected for further analysis. The ligand–protein interaction profiles were subsequently visualized and interpreted using Discovery Studio Visualizer (http://163.15.166.20:9944/DS/), which enabled the generation of 2D interaction diagrams to identify key binding residues and hydrogen bond interactions.

### 2.4 Statistical analysis

To compare *ESPL1* gene expressions between tumor and normal breast tissue samples, appropriate statistical tests were applied using curated datasets from online platforms. Log-rank tests were used to evaluate differences in survival outcomes, as well as to assess expression heterogeneity and functional enrichment patterns between high and low *ESPL1* expression groups. P-values less than 0.05 (P < 0.05) were considered statistically significant. All analyses regarding prognostic performance, expression stratification, and interaction networks were performed using statistical tools integrated within the respective online databases, ensuring methodological reproducibility and robust data interpretation.

## 3 Results

### 3.1 *ESPL1* expression in pan-cancer and BC

**FIGURE 1:**
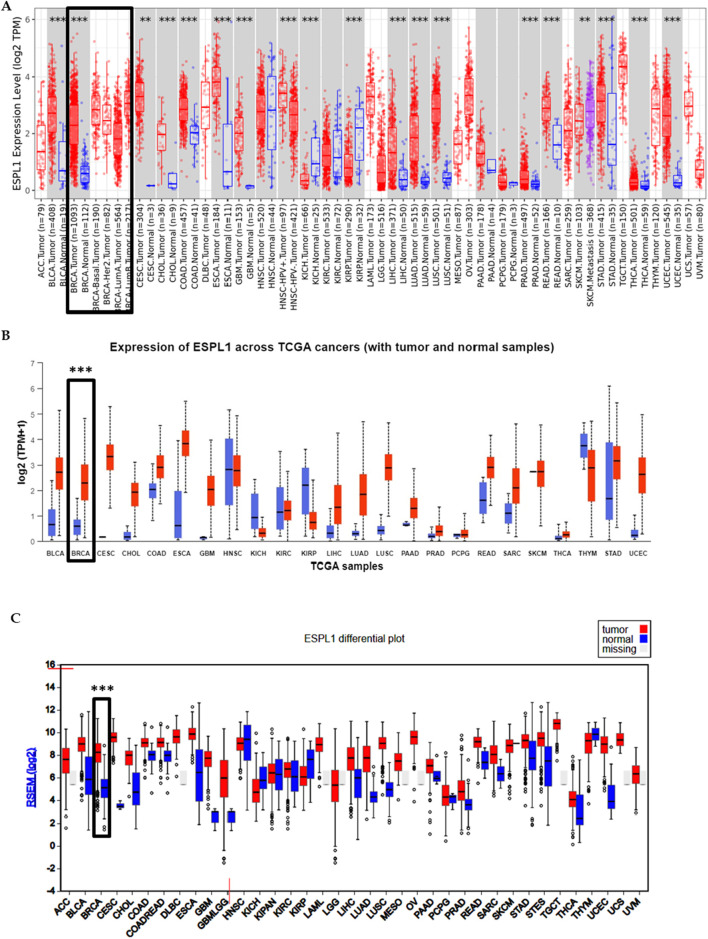
Expression pattern of *ESPL1* across pan-cancer types. **(A)** Differential expression analysis of *ESPL1* using the TIMER 2.0 database. Tumor samples (red bar-dot plot) were compared against matched normal tissues (blue bar-dot plot). **(B)** *ESPL1* expression in tumor versus normal tissues across various cancer types using the UALCAN database. Red bars represent tumor tissues; blue bars represent normal tissues. **(C)** Expression profile of *ESPL1* across multiple cancers retrieved from the FIREHOSE database, illustrating consistent overexpression in several tumor types.

**FIGURE 2:**
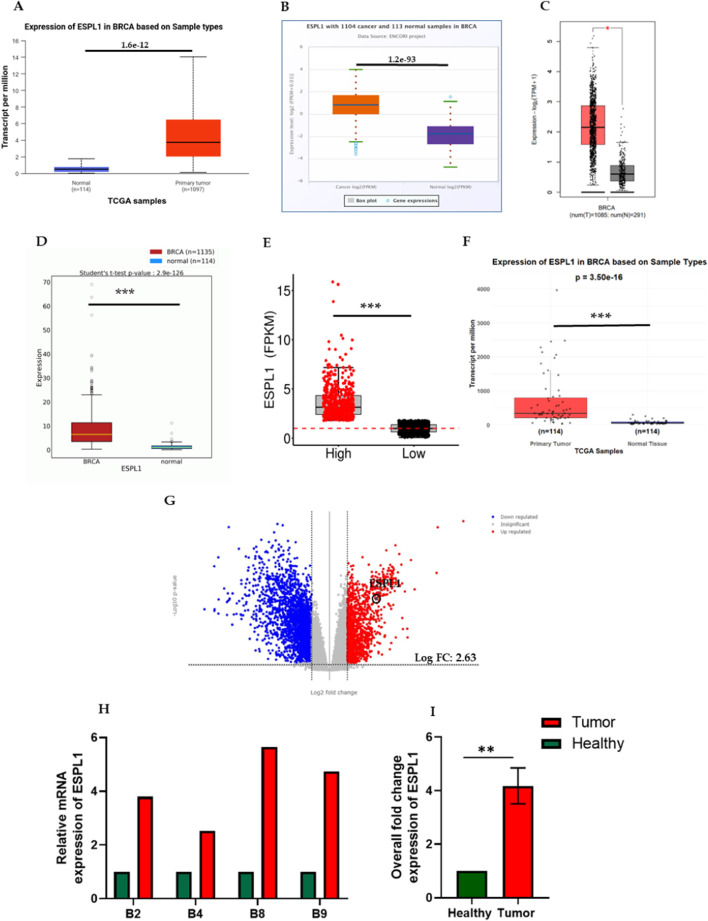
Expression of *ESPL1* in breast cancer. **(A–F)** Comparative mRNA expression of *ESPL1* in normal breast tissues and primary tumors using multiple public datasets: **(A)** UALCAN (normal *n* = 114, tumor *n =* 1,097), **(B)** ENCORI (normal *n =* 113, tumor *n =* 1,104), **(C)** GEPIA2 (normal *n =* 291, tumor *n =* 1,085), **(D)** OncoDB, **(E)** TCGA Portal, and **(F)** DESeq2 analysis of TCGA-BRCA samples in R. **(G)** Transcriptomic visualization using TCGAnalyzeR. qRT-PCR results showing **(H)** Relative mRNA expression of *ESPL1* in the primary tumor and adjacent healthy tissue of breast cancer patients. **(I)** Overall fold change expression of *ESPL1*in breast cancer patients. ** indicates p < 0.01 (p = 0.0032).

First, we analyzed the expression of *ESPL1* across all cancers included in the TCGA, including BC, using publicly available web-based tools such as TIMER 2.0, UALCAN, FIREHOSE, and OncoMX. The results revealed that *ESPL1* expression was significantly elevated in most cancer types compared to their corresponding normal tissues (Figures 1A–C; Supplementary Table S2). Next, focusing specifically on BC, we used the UALCAN database to compare *ESPL1* expression in tumor vs. normal breast tissues. As shown in Figure 2A, *ESPL1* was nearly 9-fold upregulated in BC tissues (P < 1.6e-12). This overexpression trend was consistently observed across multiple databases including ENCORI (P = 1.2e–93), GEPIA2 (P < 0.05), OncoDB (P = 2.9e–126), and the TCGA portal (P < 0.05) (Figures 2B–E).

Furthermore, *ESPL1* expression was analyzed in an equal number of normal and tumor samples (*n* = 114 each), and the results confirmed significantly higher *ESPL1* expression in tumor tissues (Figure 2F). To assess *ESPL1* expression at the transcript level in BC we used the TCGAnalyzeR database, which is based on high-end single-cell RNA sequence transcriptomic data and found a 2.63 log fold change in its expression levels (Figure 2G). Given that certain reference or housekeeping gene, maintain stable expression under most conditions and serve as internal controls in cancer transcriptomic studies, we next compared *ESPL1* expression with such genes using the CancerSEA database. This comparison further validated the upregulation of *ESPL1* in BC samples (Supplementary Figure S1A). Moreover, to validate our In-silico findings, total RNA was isolated from tissue samples of all the 8 BC patients enrolled in the study. Following RNA quality assessment, only 4 matched pairs of tumor and adjacent normal tissues were deemed suitable for downstream analysis. Subsequent qRT-PCR-based gene expression profiling revealed a significant upregulation of *ESPL1* mRNA in breast tumor tissues compared to their adjacent healthy counterparts (Figure 2H), with an overall average increase of 4-fold (Figure 2I).

### 3.2 The expression level of *ESPL1* correlates with hormone receptor status and aggressive BC subtypes

To further investigate the expression pattern of *ESPL1* across various breast cancer (BC) subtypes, we utilized the bc-GenExMiner v5.0 database, stratifying the data based on estrogen receptor (ER), progesterone receptor (PR), and triple-negative breast cancer (TNBC) status. *ESPL1* expression was found to be significantly elevated in the more aggressive BC subtypes, including ER–versus ER+, PR–versus PR+, and TNBC versus non-TNBC, as well as in basal-like versus non-basal-like tumors (Supplementary Figures S1B–D). Further subgroup analysis revealed consistently higher *ESPL1* expression in TNBC and basal-like patients compared to their non-TNBC and non-basal-like counterparts, respectively (Supplementary Figures S1E–G). We then assessed *ESPL1* expression across molecular subtypes, such as, Luminal A, Luminal B, HER2-enriched, and Basal-like and observed notably higher expression in Luminal B and Basal-like subtypes (Supplementary Figures S1H,I). Moreover, using the UALCAN database, we evaluated *ESPL1* expression across pathological stages and found it to be significantly upregulated in tumor samples across all stages compared to normal tissue (Supplementary Figure S1J). Collectively, these findings suggest that *ESPL1* overexpression is associated with aggressive BC phenotypes, highlighting its potential role as a marker of poor prognosis.

### 3.3 The role of *ESPL1* in metastasis, prognosis, and co-expression analysis

**FIGURE 3:**
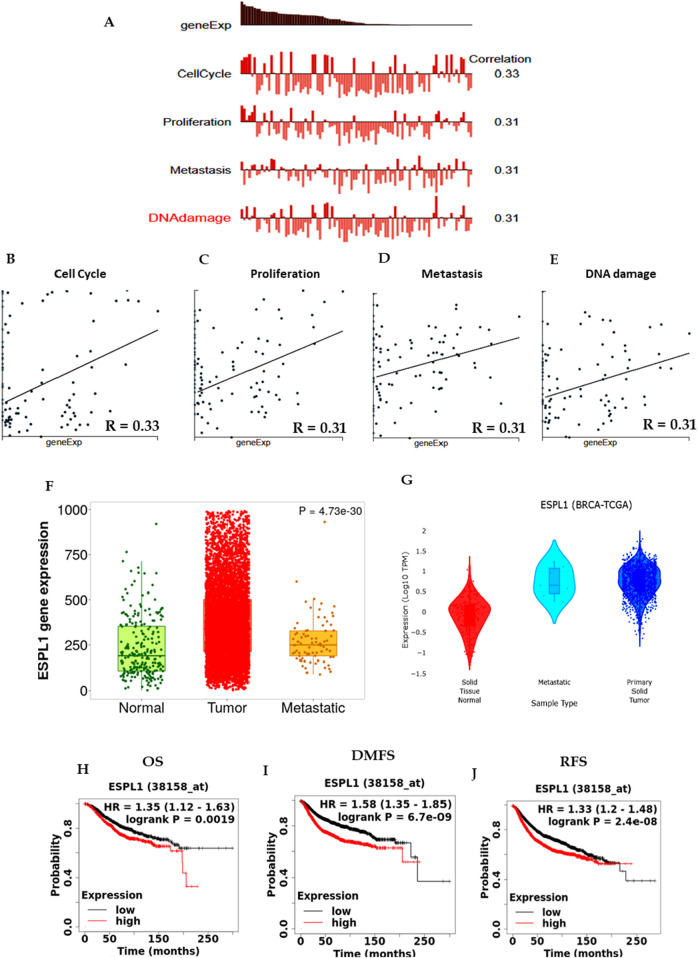
*ESPL1* expression in tumors from BC patients with biological processes, metastasis, and survival status. **(A–E)** *ESPL1* expression in biological processes using CancerSEA; **(F,G)** Metastasis in *ESPL1* expression normal, tumor, and metastasis in BC patients using the Gene Chip using TNM plot and DriverDB database. **(H–J)** Kaplan-Meier survival curves were plotted for **(H)** OS (*n =* 1879), **(I)** DMFS (*n =* 2,765), and **(J)** RFS (*n =* 4,929).

To investigate the functional relevance of *ESPL1* in breast cancer (BC), we explored its involvement in key oncogenic processes using the CancerSEA database. The results revealed that *ESPL1* expression was significantly associated with critical cancer-related pathways including cell cycle (R = 0.33), proliferation (R = 0.31), metastasis (R = 0.31), and DNA damage response (R = 0.31) (Figures 3A–E). Then, we were interested in finding out the role of *ESPL1* in metastasis. Using the TNMplot database based on RNA-Seq data, we compared *ESPL1* expression across normal tissue, primary tumors, and metastatic lesions. We observed markedly higher *ESPL1* expression in metastatic tissues, with P-value of 4.73e-30, compared to normal tissue. This finding was further validated using the DriverDBv4 platform, which confirmed a significant upregulation of *ESPL1* in metastatic samples (P = 0.00078) (Figures 3F,G). To assess the prognostic potential of *ESPL1* in BC, we performed Kaplan–Meier survival analyses using the KM Plotter database. Stratification of patients based on high and low *ESPL1* expression revealed a strong correlation between elevated *ESPL1* levels and poor clinical outcomes. Specifically, high *ESPL1* expression was associated with reduced overall survival (OS) (HR = 1.35, 95% CI: 1.12–1.63, P = 0.0019), distant metastasis-free survival (DMFS) (HR = 1.58, 95% CI: 1.35–1.85, P = 6.7e-09), and relapse-free survival (RFS) (HR = 1.33, 95% CI: 1.2–1.48, P = 2.4e-08) (Figures 3H–J; Supplementary Table S3). These findings establish *ESPL1* as a potential prognostic biomarker indicative of poor outcomes in BC patients.

Next, we examined the co-expressed genes of *ESPL1* using the Enrichr database. Ten genes: *AURKB*, *FOXM1*, *GTSE1*, *HJURP*, *KIF18B*, *KIF2C*, *KIFC1*, *PLK1*, *RRM2*, and *TROAP* were found to be significantly co-expressed with *ESPL1*. Validation via the TIMER database demonstrated a strong positive correlation (P < 0.05) between *ESPL1* and these genes, with correlation coefficients ranging from R = 0.754 to 0.880 (Table 1; Supplementary Figures S2A–J). Further analysis using the GSCA database showed that all ten co-expressed genes were overexpressed in BC, except *KIF18B*, *HJURP*, and *GTSE1* (Supplementary Figure S2K). A combined gene set variation analysis (GSVA) revealed a significantly higher expression profile of the *ESPL1* co-expression module in tumors compared to healthy tissue (Supplementary Figure S2L). Notably, these co-expressed genes also showed strong enrichment in TNBC and basal-like subtypes of BC, reinforcing their association with aggressive disease phenotypes (Supplementary Figure S2M).

**TABLE 1:** *ESPL1* co-expressed genes.

S. no	Gene	Co-expressed gene	P-value	R
1	*ESPL1*	AURKB	2.50e-231	0.785
2		FOXM1	1.54e-297	0.842
3		GTSE1	4.68–267	0.818
4		HJURP	9.20e-285	0.832
5		KIF18B	0.00e+00	0.880
6		KIF2C	1.92e-289	0.836
7		KIFC1	2.14e-289	0.836
8		PLK1	0.00e+00	0.846
9		RRM2	4.19e-203	0.754
10		TROAP	0.00e+00	0.879

### 3.4 Transcriptional regulation of *ESPL1* by E2F family members

**FIGURE 4:**
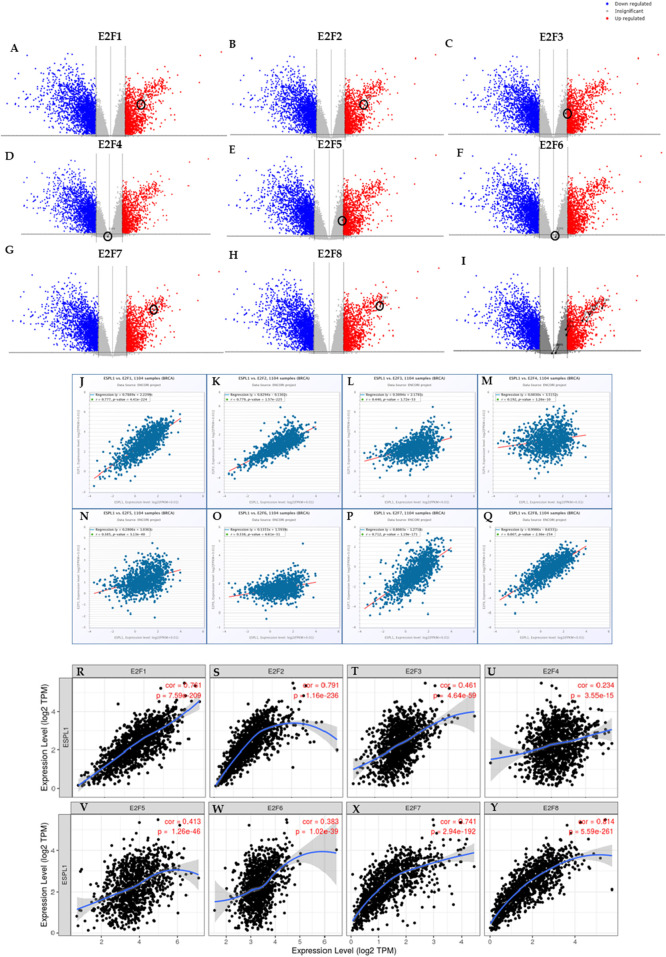
**(A–H)** Transcriptome analysis of individual E2Fs in BC using TCGA Analyzer v1.0 **(I)** *ESPL1* and E2Fs family combined expression using TCGA Analyzer v1.0 **(J–Q)** Correlation between the *ESPL1* and E2Fs using the ENCORI database **(R–Y)** Correlation between the *ESPL1* and E2Fs using the TIMER database.

Transcription factors (TFs) are critical regulators of gene expression and play essential roles in controlling diverse cellular processes such as cell cycle progression, DNA replication, apoptosis, and differentiation. Understanding the transcriptional regulation of oncogenic drivers like *ESPL1* is therefore crucial for uncovering potential molecular mechanisms underlying cancer aggressiveness and for identifying novel therapeutic targets. To investigate the transcriptional regulators of *ESPL1*, Enrichr database was used to identify related pathways and found that E2F targets were significantly associated with *ESPL1* as shown in Table 2. We focused on the E2F family of transcription factors, as they are known for their central role in controlling the G1/S transition of the cell cycle and promoting the transcription of genes involved in mitotic progression. This family comprises eight members (*E2F1*–*E2F8*), which function either as transcriptional activators or repressors depending on cellular context. Furthermore, Using TCGAnalyzeR, we analyzed the expression profiles of E2F genes in BC tumors and found a significant upregulation of *E2F1*, *E2F2*, *E2F7*, and *E2F8*, with log2 fold-change values of 2.09, 2.41, 2.94, and 3.49, respectively (Figures 4A–H). In contrast, *E2F3*, *E2F4*, *E2F5*, and *E2F6* showed minimal or no significant change in expression (0.88, −0.11, 0.94, and 0.14, respectively). A combined expression plot (Figure 4I) demonstrated that *E2F7* and *E2F8* were in closest proximity to *ESPL1*, suggesting a potential regulatory axis between these TFs and *ESPL1*, which itself was upregulated with a log2 fold-change of 2.63.

**TABLE 2:** *ESPL1* associated pathways.

S. no	Name	P-value	Adjusted P-value	Odds ratio	Combined score
1	E2F Targets	2.301e-8	10.381e-8	101.51	1785.33
2	G2-M Checkpoint	0.000001944	0.000005832	67.33	885.39
3	Mitotic Spindle	0.004206	0.006371	25.12	137.43
4	mTORC1 Signalling	0.004247	0.006371	24.99	136.48
5	Myc Targets V2	0.02863	0.03436	38.86	138.07
6	Spermatogenesis	0.06550	0.06550	16.46	44.88

To corroborate this potential relationship, we performed correlation analyses using the ENCORI and TIMER databases. Interestingly, ENCORI analysis confirmed that all E2Fs were significantly correlated with *ESPL1* expression in BC (Figures 4J–Q). The TIMER analysis further supported these findings and highlighted only *E2F7* (R = 0.741) and *E2F8* (R = 0.814) as having the strongest positive correlations with *ESPL1* among the E2F members (Figures 4R–Y). Together, these results suggest that *E2F7* and *E2F8* may act as critical upstream transcriptional regulators of *ESPL1*, potentially promoting its overexpression in aggressive subtypes of breast cancer.

### 3.5 Post-transcriptional regulation of *ESPL1* expression by miRNAs

While the transcriptional regulation of *ESPL1* in breast cancer (BC) is increasingly understood, the mechanisms underlying its aberrant upregulation remain incompletely elucidated. Given the prominent role of microRNAs (miRNAs) in post-transcriptional gene silencing, we sought to identify miRNAs potentially involved in the negative regulation of *ESPL1* expression in BC. We utilized the miRNet platform to construct a comprehensive miRNA–*ESPL1* interaction network, identifying the top 11 miRNAs predicted to regulate *ESPL1* (Supplementary Figure S3A). Further, the expression data from the UALCAN database revealed that four miRNAs, *hsa-miR-10a-5p*, *hsa-let-7b-5p*, *hsa-miR-214-3p*, and *hsa-miR-1-3p* were significantly downregulated in tumor tissues from BC patients (Supplementary Table S4). Among these, *hsa-let-7b-5p* and *hsa-miR-10a-5p* exhibited a strong negative correlation with *ESPL1* expression, analyzed using the ENCORI database. To assess the prognostic value of these miRNAs, we analyzed survival plots using the CancerMIRNome database. Although *hsa-miR-10a-5p* showed a tumor suppressive expression pattern, it was not significantly associated with survival outcomes in BC patients (Supplementary Figure S3B). Intrestingly, *hsa-let-7b-5p* emerged as a key tumor suppressor miRNA, not only did it negatively correlate with *ESPL1* expression, but it also demonstrated strong associations with transcriptional regulators, particularly *E2F8*, in the ENCORI database (Supplementary Figures S3C,D), suggesting a regulatory axis involving *hsa-let-7b-5p*, *E2F8*, and *ESPL1*.

**FIGURE 5:**
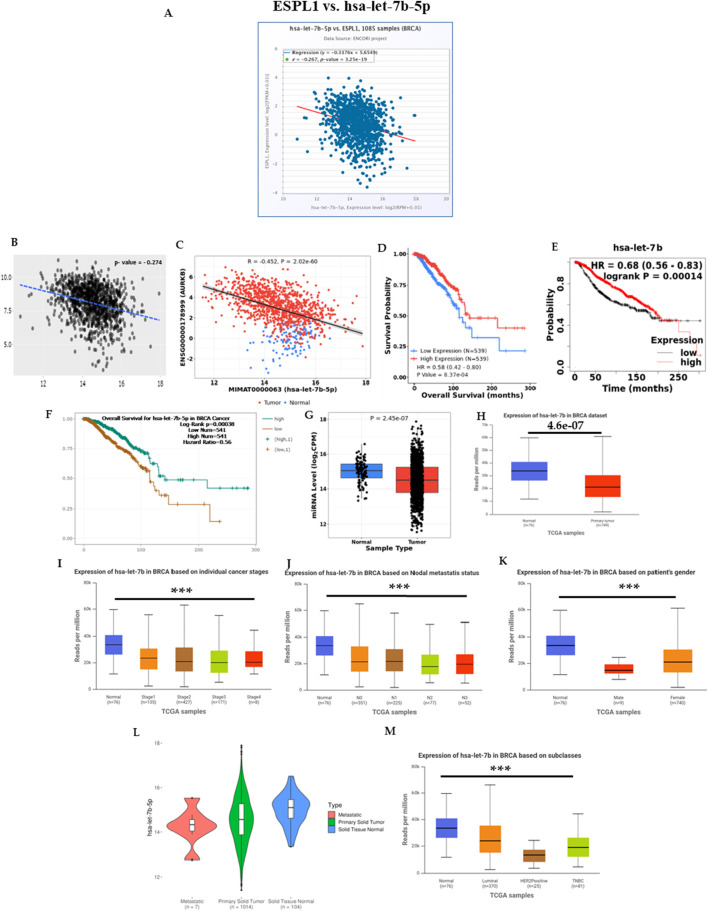
miRNA expression correlation with *ESPL1* in tumor tissues from BC patients was determined by using the ENCORI, TACCO, and CancerMIRNome databases (**A–C**, respectively). **(A)** Boxplot of correlation between let-7b-5p and *ESPL1*; **(B)** Transcriptome analysis using TACCO; **(C)** correlation between the *ESPL1* and let-7b-5p using CancerMIRNome; **(D–F)** *hsa-let-7b-5p* survival status by using CancerMIRNome, KM Plotter, and ENCORI; **(G)** let-7b-5p expression in tumor vs. normal using CancerMIRNome; **(H)** Boxplot of let-7b-5p expression in BC (*n =* 149) vs. normal (*n =* 78) using UALCAN; **(I)** Boxplot of let-7b-5p, expression according to normal versus tumor stages (1, 2, 3, and 4) by UALCAN; **(J)** Boxplot of let-7b-5p expression according to normal versus tumor in BC patients with different nodal statuses (N0, N1, N2, and N3) **(K)** Boxplot of let-7b-5p, expression according to normal versus male and female. **(L)** Metastasis expression in miRNA let-7b-5p metastatic, primary solid tumor, and solid tissue normal in BC patients using UALCAN. **(M)** Boxplot of let-7b-5p expression according to normal versus tumors from patients with different histological subtypes (luminal, HER2+, and TNBC).

We further validated the negative correlation between *hsa-let-7b-5p* and *ESPL1* using ENCORI, TACCO, and CancerMIRNome datasets, all of which showed statistically significant negative relationships (Figures 5A–C). Additionally, Kaplan–Meier survival analyses using CancerMIRNome (HR = 0.58, P = 8.37e-04), KM Plotter (HR = 0.68, P = 0.00014), and ENCORI (HR = 0.56, P = 0.00014) consistently indicated that low expression of *hsa-let-7b-5p* is significantly associated with poor prognosis in BC patients (Figures 5D–F). Expression profiling of *hsa-let-7b-5p* across tumor and normal tissues in BC patients revealed significant downregulation in tumors using CancerMIRNome (P = 2.45e-07) and UALCAN (P = 4.6e-07) as shown in Figures 5G,H. Further stratification by clinical parameters assessed using UALCAN demonstrated that *hsa-let-7b-5p* expression was reduced in advanced pathological stages, nodal metastasis, and across both male and female patients (Figures 5I–K). Analysis through ExplORRnet confirmed its downregulation in metastatic BC patients (Figure 5L), while UALCAN analysis indicated lower expression in triple-negative breast cancer (TNBC) cases (Figure 5M), reinforcing its relevance in aggressive disease subtypes. Together, these findings implicate *hsa-let-7b-5p* as a critical post-transcriptional regulator of *ESPL1* and highlight a potential ceRNA network involving *hsa-let-7b-5p*, *E2F8*, and *ESPL1* that may drive tumor progression and poor prognosis in breast cancer.

### 3.6 Regulation of *ESPL1* expression is mediated by *TMPO-AS1* and *let-7b-5p*

**FIGURE 6:**
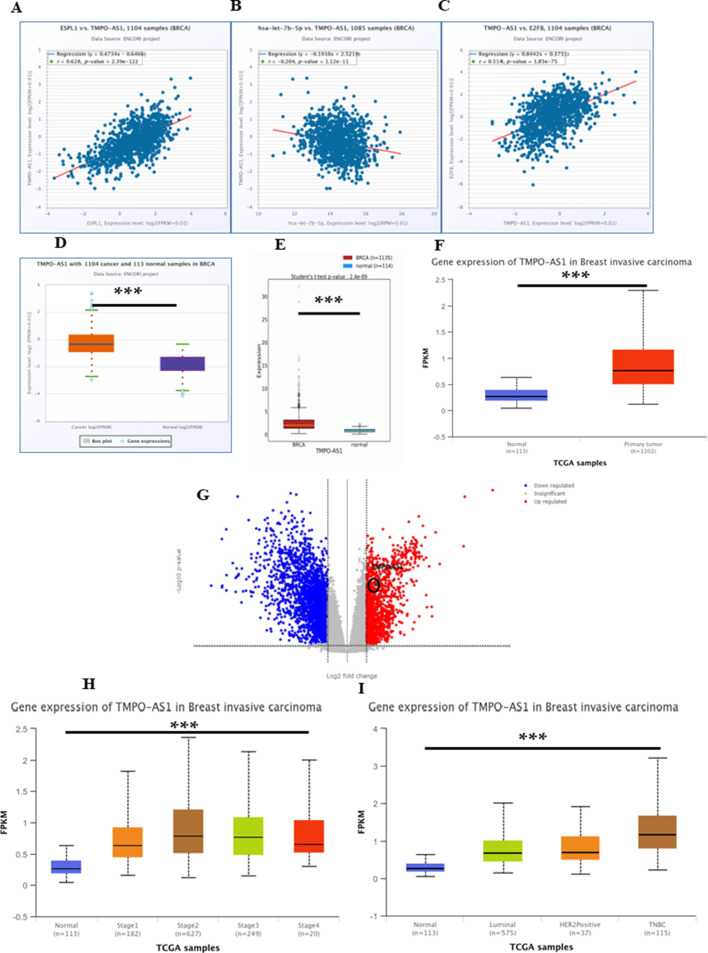
**(A–C)** ENCORI database showing **(A)** Correlation between *TMPO-AS1* and *ESPL1*, **(B)** Correlation between *TMPO-AS1* and let-7b-5p, **(C)** Correlation between *TMPO-AS1* and *E2F8* **(D)** Boxplot of *TMPO-AS1* expression (*n =* 1,104) and normal (*n =* 113) BC samples using the ENCORI database; **(E)** OncoDB normal vs. tumor; **(F)** Boxplot *TMPO-AS1* gene expression in normal vs. tumor using UALCAN; **(G)** Transcriptome analysis of *TMPO-AS1* using TCGA AnalyzeR v1.0; **(H)** Boxplot of *TMPO-AS1*, expression according to normal versus tumor stages (1, 2, 3, and 4); by UALCAN; **(I)** Boxplot of *TMPO-AS1*, expression according to normal versus tumors from patients with different histological subtypes (Luminal, HER2+, and TNBC).

In cellular systems, lncRNAs, miRNAs, and mRNAs can form ceRNA networks, collaboratively modulating gene expression and cellular processes. To identify lncRNAs that might modulate *ESPL1* expression via interaction with *hsa-let-7b-5p*, we used the Enrichr database, and several candidate lncRNAs were identified to be associated with *ESPL1*, including *LINC00618*, *SGO1-AS1*, *DEPDC1-AS1*, *LIX1L-AS1*, and *LINC01775* (Table 3). Further, using the UALCAN database, we found that *DDX11-AS1*, *TMPO-AS1*, *DEPDC1-AS1*, and *CSRP3-AS1* were upregulated in BC tissues compared to normal controls. Among them, ENCORI analysis revealed that *TMPO-AS1* had the strongest positive correlation with *ESPL1* (R = 0.628, P = 2.39e-122) (Supplementary Table S5; Figure 6A). Notably, *TMPO-AS1* showed a significant negative correlation with *hsa-let-7b-5p* (R = −0.204, P = 1.12e-11), suggesting a ceRNA-based regulatory relationship (Figure 6B). Given our earlier findings that *E2F8* might regulate *ESPL1*, we further assessed its correlation with *TMPO-AS1*. ENCORI analysis showed a strong positive association between *TMPO-AS1* and *E2F8* (R = 0.514, P = 1.85e-75) (Figure 6C), further supporting their interaction within a regulatory axis. We validated the expression levels of *TMPO-AS1* using multiple datasets. lncRNADisease v3.0 was used to assess the pan-cancer expression profile of *TMPO-AS1* and it was found to be overexpressed amongst several malignancies with Breast Neoplasms at the top (Supplementary Table S6). Further, ENCORI, OncoDB, UALCAN, and TCGAnalyzeR databases consistently demonstrated significantly higher expression of *TMPO-AS1* in BC tumors compared to normal tissues (Figures 6D–G). Moreover, UALCAN data showed *TMPO-AS1* expression was elevated across different pathological stages and was also upregulated in TNBC compared to normal controls (Figures 6H,I). Additionally, using miRNet database and a direct *TMPO-AS1*/*hsa-let-7b-5p*/*ESPL1* axis was revealed supporting a ceRNA model whereby *TMPO-AS1* sponges *hsa-let-7b-5p*, thereby releasing repression on *ESPL1* (Supplementary Figure S3E). To further illustrate these regulatory interactions, a lncRNA–miRNA–mRNA network was constructed using Cytoscape (3.10.3). The resulting visualization highlighted a potential regulatory cascade involving TMPO-AS1, hsa-let-7b-5p, E2F8, and ESPL1, with directional arrows indicating the predicted regulatory flow (Supplementary Figure S3F).

**TABLE 3:** *ESPL1* associated lncRNAs.

S. no.	Name	P-value	Adjusted P-value
1	PRC1-AS1	6.140e-24	5.526e-22
2	CSRP3-AS1	1.309e-17	2.356e-16
3	H2AZ1-DT	1.309e-17	2.356e-16
4	DDX11-AS1	1.309e-17	2.356e-16
5	*TMPO-AS1*	1.309e-17	2.356e-16
6	LINC00618	7.476e-15	8.410e-14
7	SGO1-AS1	7.476e-15	8.410e-14
8	DEPDC1-AS1	7.476e-15	8.410e-14
9	LIX1L-AS1	2.772e-12	2.268e-11
10	LINC01775	2.772e-12	2.268e-11

Furthermore, to assess this regulatory potential of *hsa-let-7b-5p* on key oncogenic drivers, interaction analyses were conducted with *ESPL1* (NM_012291.5), *E2F8* (NM_001256371), and the lncRNA *TMPO-AS1* (NR_027157.1) (Table 4). miRWalk predicted strong binding affinities of *hsa-let-7b-5p* to *ESPL1* and *E2F8*, with minimum free energy (MFE) values of −24.4 kcal/mol and −22.2 kcal/mol, respectively. RNA22v2 folding energy analysis corroborated these findings, showing energetically favorable heteroduplex formations with *ESPL1* (−22.0 kcal/mol), *E2F8* (−14.4 kcal/mol), and *TMPO-AS1* (−12.4 kcal/mol). Additionally, the heteroduplex modeling revealed specific base pairing between *hsa-let-7b-5p* and each transcript, reinforcing the likelihood of direct post-transcriptional regulation. Collectively, these data suggest that *hsa-let-7b-5p* may suppress oncogenic mRNAs *ESPL1* and *E2F8*, key cell cycle regulators, while its interaction with the oncogenic lncRNA *TMPO-AS1* indicates a ceRNA mechanism. *TMPO-AS1* likely sequesters *hsa-let-7b-5p*, relieving repression of *ESPL1* and *E2F8*, thus, upregulation of *hsa-let-7b-5p* could disrupt this ceRNA network, attenuating cancer progression by simultaneously targeting oncogenic transcripts and their sponge lncRNA.

**TABLE 4:** Binding affinities of *hsa-let-7b-5p* with *ESPL1*, *E2F8* and *TMPO-AS1*.

miRNA	Transcript	Binding energy (miRWalk)	Folding energy (RNA22v2) (in -Kcal/mol)	Heteroduplex
*hsa-let-7b-5p* MIMAT0000063	*ESPL1* NM_012291.5	−24.4	−22.0	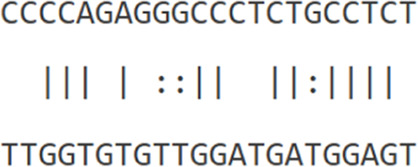
	*E2F8* NM_001256371	−22.2	−14.40	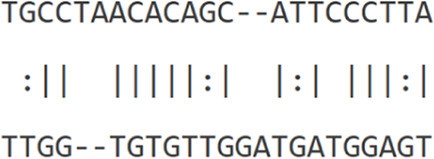
	*TMPO-AS1* NR_027157.1	-	−12.40	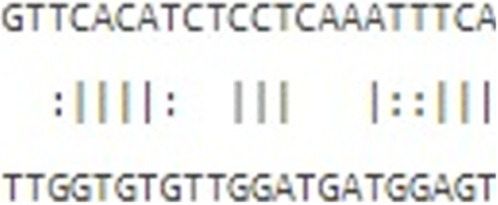

### 3.7 Mechanism of *ESPL1* dysregulation in BC

To develop effective therapeutic strategies for ER-/PR-breast cancers, it is essential to understand the molecular mechanisms underlying *ESPL1* overexpression. Our analysis identified a significant regulatory axis involving *TMPO-AS1*, which showed a strong positive correlation with the proliferation marker *MKI67* (Supplementary Table S6). *MKI67* is widely used in pathological evaluations and is tightly associated with tumor cell proliferation and growth (Li et al., 2014). To establish the relation of *MKI67* with our target genes ccorrelation analyses were performed using ENCORI database which revealed consistent co-expression patterns between *MKI67* and *ESPL1* (R = 0.827), *E2F8* (R = 0.816), *hsa-let-7b-5p* (R = −0.153), and *TMPO-AS1* (R = 0.540) in breast cancer and the same correlations were corroborated (Supplementary Figures S4A–G). Furthermore, we explored the relationship between these genes and the expression of estrogen receptors (*ESR1*) and progesterone receptors (*PGR*), both of which are key biomarkers and therapeutic targets in BC.

**FIGURE 7:**
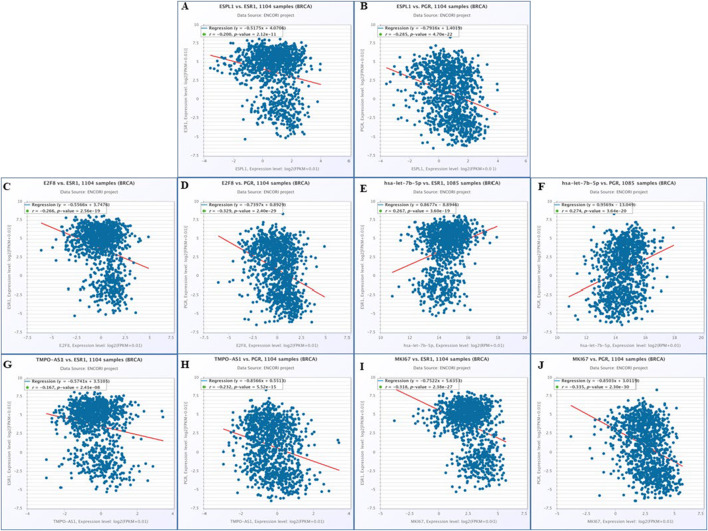
Molecular mechanism of regulatory network associated with *ESR1* and *PGR* genes by using ENCORI. Correlation between **(A)** *ESPL1* vs. *ESR1*; **(B)** *ESPL1* vs. *PGR*; **(C)** *E2F8* vs. *ESR1*; **(D)** *E2F8* vs. *PGR* **(E)** has-let-7b-5p vs. *ESR1* **(F)** has-let-7b-5p vs. *PGR* **(G)** *TMPO-AS1* vs. *ESR1* **(H)** *TMPO-AS1* vs. *PGR* **(I)** *MKI67* vs. *ESR1* **(J)** and *MKI67* vs. *PGR* were evaluated.

Using the ENCORI database, *ESR1* and *PGR* exhibited significant negative correlations with *ESPL1* (R = −0.200, P = 2.12e-11; R = −0.258, P = 4.70e-22) (Figures 7A,B), while *E2F8* similarly negatively correlated with *ESR1* (R = −0.266, P = 2.56e-19) and *PGR* (R = −0.329, P = 2.40e-29) (Figures 7C,D). In contrast, *hsa-let-7b-5p* positively correlated with both *ESR1* (R = 0.267, P = 3.60e-19) and *PGR* (R = 0.274, P = 3.64e-20) (Figures 7E,F). *TMPO-AS1* was inversely correlated with *ESR1* (R = −0.167, P = 2.41e-08) and *PGR* (R = −0.232, P = 5.52e-15) (Figures 7G,H). *MKI67* also showed strong negative correlations with *ESR1* (R = −0.318, P = 2.38e-27) and *PGR* (R = −0.335, P = 2.30e-30) (Figures 7I,J). We did the cross-validation of the correlation values using OncoDB and found the correlation patterns (Supplementary Figures S4H–O). Consistent with these observations, expression profiling demonstrated that *ESPL1*, *E2F8*, and *MKI67* were overexpressed across BC stages, while *ESR1* and *PGR* were downregulated, particularly in more aggressive or hormone receptor-negative subtypes. Notably, *ESPL1* expression patterns closely mirrored those of *MKI67*, as shown in Supplementary Figure S4P. To our knowledge, this is the first *in-silico* study to report *ESPL1* overexpression mimicking *MKI67* expression in breast cancer. These findings suggest that elevated *ESPL1* expression could serve as a proxy for tumor cell proliferation, particularly in ER-/PR-subtypes and intermediate stages of disease. Thus, *ESPL1* emerges as a promising marker of aggressive tumor growth and potential therapeutic vulnerability in hormone receptor-negative breast cancers.

### 3.8 Docking analysis

**FIGURE 8:**
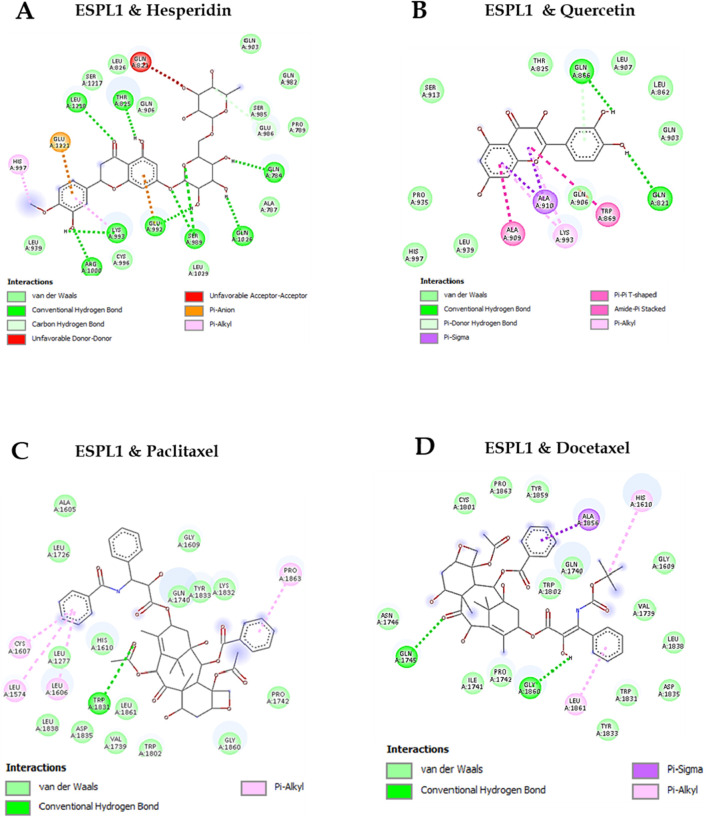
2D Visualization of *ESPL1* with docked compounds. **(A)** Hesperidin, **(B)** Quercetin, **(C)** Paclitaxel, **(D)** Docetaxel.

Molecular docking studies were conducted to evaluate the binding affinities of natural and chemotherapeutic compounds with *ESPL1*. Among the tested ligands, Hesperidin exhibited the binding affinity with *ESPL1* at −10.8 kcal/mol and Quercetin demonstrated the binding affinity of −7.8 kcal/mol indicating a strong and stable interaction. In comparison, Paclitaxel and Docetaxel showed binding affinities of −8.4 kcal/mol and −8.2 kcal/mol, respectively (Table 5). The highest docking score of Hesperidin suggests that it may serve as a potent inhibitor of *ESPL1*. These interactions were further supported by 2D molecular interaction diagrams generated in Discovery Studio Visualizer (Figures 8A–D). The combination of multiple hydrogen bonds, strong van der Waals contacts, and favorable π-alkyl interactions with key active site residues such as GLU432, GLN406, and HIS397 implies that Hesperidin not only fits tightly within the *ESPL1* binding site but may also effectively inhibit its activity. In contrast, chemotherapeutic agents demonstrated fewer stabilizing interactions within the binding pocket. Notably, the extensive hydrogen bonding and π-π stacking of Hesperidin suggest a more stable and specific binding to *ESPL1*, highlighting its potential as a promising inhibitor.

**TABLE 5:** Binding affinities of *ESPL1* with therapeutic targets.

Molecule	Binding affinity			
	Hesperidine	Quercetine	Paclitaxel	Docetaxel
*ESPL1*	−10.8 kcal/mol	−7.8 kcal/mol	−8.4 kcal/mol	−8.2 kcal/mol

## 4 Discussion

Breast Cancer is a multifactorial disease, with recurrence and drug resistance being the primary causes of mortality (Smolarz et al., 2022). The heterogeneous subtypes of BC respond differently to therapies, resulting in different outcomes. Conventional clinical and pathological classifications do not fully capture the complexity of this disease, making them limited in therapeutic decisions and prognosis. Thus, it is crucial to identify unique prognostic signature patterns that can help in the early intervention for disease progression, recurrence or metastasis. In this regard, dysregulated cell cycle is one of the hallmarks of cancer; herein, extra spindle pole bodies-like 1 (*ESPL1*) is a cysteine endopeptidase or separase that helps sister chromatids stick together before and separate at the right time during anaphase (Nie et al., 2022). Therefore, constitutive activation of *ESPL1* can lead to aneuploidy, DNA damage, and the loss of crucial tumor suppressor gene sites, which are associated with tumor growth and disease progression (Zhang Y. et al., 2024). For example, overexpression of *ESPL1* in the mammary glands of MMTV-*ESPL1* mice causes them to form aggressive mammary adenocarcinomas with high levels of genetic instability, cell cycle defects, poor differentiation, distant metastasis and metaplasia (Mukherjee et al., 2014). In addition, abnormal expression of *ESPL1* in endometrial cancer (EC) facilitates metastasis and invasion, leading to a poor prognosis (Yang et al., 2024). *ESPL1* also participates in the occurrence and development of other human cancers, which is associated with reduced patient survival. However, the regulatory mechanisms of *ESPL1* in BC are not fully explored. At the experimental level, our study found that *ESPL1* expression was higher in BC tissues compared to normal breast tissues. This was further corroborated using *in silico* databases, wherein we found a strong association between the higher expression of *ESPL1* in tumors and worse outcomes in BC patients, especially those with low-grade BCs. The biological behavior of many tumors, including metastasis and proliferation, heavily relies on *ESPL1*, necessitating further research to clarify and expand upon these findings.

Various cancer types, including BC (Finetti et al., 2014), bladder cancer (Zhang W. et al., 2024), esophageal carcinoma (Liu et al., 2021), gastric cancer (Zhang B. et al., 2024), liver cancer (Song et al., 2022), lung cancer (Nie et al., 2022), and endometrial cancer (Yang et al., 2024), exhibit elevated *ESPL1* expression. In this study, we showed the highest increase in *ESPL1* expression in tumors from TNBC patients, followed by Basal and luminal subtypes. Furthermore, our study also demonstrated a strong association with higher expression of *ESPL1* with OS, DMFS, and RFS in BC patients. Regulation of transcription factors is critical to cancer stemness, allowing cancer stem cells to maintain and function (Modi et al., 2022). Coexpression analysis showed positive correlations between ESPL1 expression and several genes, including AURKB, FOXM1, GTSE1, HJURP, KIF18B, KIF2C, KIFC1, PLK1, RRM2, and TROAP. These genes are well-known regulators of key processes in cell cycle progression, chromosomal segregation, and mitotic spindle dynamics, which are pathways directly related to ESPL1’s function as a critical regulator of chromatid separation. The strong correlations observed suggest that ESPL1 may act synergistically with these genes within the same oncogenic pathways, promoting uncontrolled proliferation and tumor progression in BC. Cells require E2F transcription factors (E2Fs) for cell division, proliferation and survival (Kassab et al., 2023). We used several computational tools to establish that *E2F7* and *E2F8* are co-expressed with *ESPL1* in BC, with *E2F8* showing the highest positive correlation.

Finetti et al. (2014) reported that *ESPL1* is linked to the aggressive biological behavior of various human tumors, promoting the development and proliferation of tumor cells and leading to poor patient outcomes (Finetti et al., 2014). Additionally, Hu et al. (2020) discovered that a fusion gene involving human *ESPL1* integrated with HBV S may serve as a potential biomarker for the early diagnosis of hepatocellular carcinoma (HCC) in patients infected with HBV (Hu et al., 2020). *ESPL1* has also been implicated in the increased malignancy of both non-small cell and small cell lung cancer, positioning it as a potential target for molecular therapy in lung cancer. Similar findings have been observed in other malignancies, including rectal adenocarcinoma, bladder cancer, and prostate carcinoma (Zhang and Pati, 2017). Recent research using CRISPR gain-of-function screening has identified several new targets associated with resistance to apatinib, including MCM2, CCND3, *ESPL1*, and PLK1. Inhibiting *ESPL1* could enhance the sensitivity of gastric cancer (GC) cells to apatinib treatment. Simultaneously, downregulating mouse double minute 2 (MDM2) could restore the sensitivity of GC cells to apatinib and counteract the resistance mediated by *ESPL1*. Recent clinical studies confirm a critical role for the *BRD4*/*ALKBH5*/*ESPL1* pathway in BC progression (Zhang et al., 2025). This research is significant in revealing the etiology of breast cancer by elucidating the function of *ESPL1*, which may provide a potential molecular marker for the diagnosis and treatment of breast cancer, particularly concerning its aggressiveness.

Several microRNAs are dysregulated during carcinogenesis, recurrence and drug resistance (Hajizadeh et al., 2023). miRNAs serve as potential biomarkers for various diseases, focusing on gene expression control, drug sensitivity, and resistance mechanisms (Elimam et al., 2024). miRNAs collaborate with mRNA, proteins, and other non-encoding RNAs to establish a regulatory network with biological functions and potential medical applications (Sideris et al., 2022). miRNA-related treatments have great potential in cancer treatment, with better efficacy and safety than siRNA-based treatments. However, we must address issues such as tumor cell heterogeneity and drug diversity. Using several databases, we identified 11 miRNAs associated with *ESPL1*, as well as four downregulating miRNAs. The miRNet platform discovered a negative correlation between *hsa-let-7b-5p* and *hsa-mir-10a-5p* with *ESPL1* but did not find significant correlation of hsa-mir-10a-5p with the survival outcomes in BC (Supplementary Figure S3B). However, we found a negative association between *hsa-let-7b-5p* and *ESPLI* gene expression. In particular, *hsa-let-7b-5p* has been shown to inhibit aerobic glycolysis and metastasis in breast cancer by repressing hexokinase 2, indicating its central role in metabolic reprogramming and tumor suppression (PMID: 37019900). Lower expression of *hsa-let-7b-5p* in tumors correlate with better survival outcome of BC patients.

Further, long non-coding RNAs (lncRNAs) are a crucial group of over 200 nucleotides that play a crucial role in cancer development and pathological processes (Sideris et al., 2022). They regulate gene expression, chromatin modification, splicing, and mRNA stability, as well as interact with other RNAs and proteins (Sebastian-delaCruz et al., 2021). lncRNAs like *MALAT1*, *H19*, and *MEG3* play a big role in controlling the cell cycle by affecting *p21* or *p53* (Aravindhan et al., 2021; Hashemi et al., 2022). lncRNAs interact with miRNAs in RNA regulation, promoting gene expression and altering it in various diseases, particularly cancer (Entezari et al., 2022). They have the potential to contribute to cancer onset, modulate cancer hallmarks, and promote progression. They also play a role in epithelial-mesenchymal transition and metastasis in various tumors (Ma et al., 2022). In this study, we utilized the Enrichr database to study the impact of miRNAs on lncRNA stability. lncRNAs, namely *PRC1-AS1*, *CSRP3-AS1*, *H2AZ1-DT*, *DDX11-AS1*, *TMPO-AS1*, *LINC00618*, *SGO1-AS1*, *DEPDC1-AS1*, *LIX1L-AS1*, and *LINC01775* were found to be associated with *ESPL1*. We found five upregulated lncRNAs in BC patients, with *TMPO-AS1* showing the most significant positive correlation. We also performed network analysis to see the interaction of non-coding RNA with *ESPL1* and its co-expressed genes. This led us to the conclusion that *E2F8* might regulate *ESPL1* expression, with *TMPO-AS1* being overexpressed in BC patients and a significant rise with different stages of BC. Interestingly, we also found a very strong correlation between *TMPO-AS1* and BC, with a correlation coefficient of 0.999893 with all the subtypes of BC. Recent reports have implicated *TMPO-AS1* in various oncogenic processes in breast cancer. It functions as a ceRNA for *miR-4731-5p* and promotes *FOXM1* signaling (Wang et al., 2021), while another study showed that *TMPO-AS1* promotes chemoresistance and invasion via the *miR-1179/TRIM37* axis (Ning et al., 2021). Notably, a 2024 study demonstrated that *TMPO-AS1* sponges *miR-383-5p* to upregulate LDHA in TNBC, reinforcing its role as a ceRNA in aggressive BC subtypes (Vats et al., 2024).

Furthermore, we discovered that BC stages exhibited overexpression of *ESPL1*, *E2F8*, and *MKI67*, as well as downregulation of *ESR1* and *PGR*. Our study also found a strong negative association between the expression of *ESPL1*, *E2F8*, and *MKI67* and the genes for estrogen and progesterone receptors (ER/PR) (Supplementary Figures S4G–J). The study also examined the association between *MKI67*, *ESPL1*, *E2F8*, *hsa-let-7b-5p*, and *TMPO-AS1*. To the best of our knowledge, this is the first study to report *in silico ESPL1* overexpression mimicking estrogen and progesterone receptor gene expression in BC. High levels of *ESPL1* may cause aggressive BC or ER-negative PR-negative BC. This study also revealed that *ESPL1* gene expression is closely associated with quiescent and cancer invasion and metastasis. Building upon the regulatory insights, we further explored the therapeutic relevance of ESPL1 through molecular docking analysis. Among the tested compounds, the natural flavonoid Hesperidin exhibited the strongest binding affinity with ESPL1 (−10.8 kcal/mol), followed by Paclitaxel (−8.4 kcal/mol), Docetaxel (−8.2 kcal/mol), and Quercetin (−7.8 kcal/mol). The interaction of Hesperidin with key residues such as GLU432, GLN406, and HIS397 indicates a stable and specific binding configuration. These findings suggest that Hesperidin may function as a potential ESPL1 inhibitor and could be explored as a candidate for targeted therapeutic strategies in BC. Our results are consistent with prior studies highlighting the anticancer potential of natural compounds. For instance, Hesperidin and Quercetin have been reported to inhibit cell proliferation, induce apoptosis, and enhance the sensitivity of breast cancer cells to chemotherapeutics (Ezzati et al., 2020; Prieto-Vila et al., 2020; Hermawan et al., 2021; Shakiba et al., 2023). By integrating transcriptomic, ceRNA network, and docking-based findings, our study provides a multi-dimensional framework positioning ESPL1 as both a prognostic biomarker and a potential therapeutic target in breast cancer.

## 5 Conclusion

The *in silico* study discovered that lncRNA *TMPO-AS1* induces the *ESPL1*/*E2F8* pathway in ER/PR cells. The study also found that BC patients have nine-fold higher *ESPL1* gene expression compared to normal tissues. The study also found that E2Fs, a family of eight genes involved in cell cycle regulation, regulate *ESPL1* gene expression in BC. We explored the interaction between *ESPL1*, miRNAs, and lncRNAs, as the upregulation of *ESPL1* in BC remains a mystery. The *in silico* study found that *hsa-let-7b-5p* affects the stability of *TMPO-AS1*/*ESPL1*/*E2F8* in a sponge-like way, and that miRNAs can lower it in BC. Understanding the biological process behind *ESPL1* gene overexpression is crucial for effective treatment planning in BC.

## Data Availability

The datasets presented in this study can be found in online repositories. The names of the repository/repositories and accession number(s) can be found in the article/Supplementary Material.

## Abbreviations

bc-GenExMiner v5.0, Breast Cancer Gene Expression Miner; BC,Breast Cancer; CancerSEA, Cancer Single Cell State Atlas; CD4+T cells, Cluster of Differentiation 4 positive T cells; ceRNA, Competing Endogenous RNA; ctcRbase, Circulating Tumor Cells Database; DMFS, Distant Metastasis Free Survival; DNA, Deoxyribonucleic Acid; DNMIVD, Disease and Non-Disease Mutation Impacting Variant Database; E2F, Eukaryotic Transcription Factor; ENCORI, Encyclopaedia of RNA Interactomes; ER, Estrogen Receptor; *ESPL1*, Extra Spindle Pole Bobies Like 1; GEPIA2, Gene Expression Profiling Interactive Analysis; GSCA, Gene Set Cancer Analysis; Has-let-7b, Homosapiens MicroRNA Family; HER2, Human Epidermal Growth Factor Receptor 2; KM PLOTTER, Kaplan-Meier Plotter; LncRNA, long noncoding RNA; MiRNA, Micro Ribonucleic Acid; *MKI67*, Marker of Proliferation Ki-67; OS, Overall Survival; PR, Progesterone Receptor; RFS, Relapses Free Survival; RNA, Ribonucleic Acid; TACCO, Transcriptome Alterations in Cancer Omnibus; TCGA Portal, The Cancer Genomic Atlas Portal; TCGAnalyzerv1.0, The Cancer Genome Altas Analyzer; TIICs, Tumor Infiltration Immune Cells; TIMER 2.0, Tumor Immune Estimation Resource; TISIDB, Tumor and Immune System Interaction Database; *TMPO-AS1*, Thymopoietin Antisense Transcript1; TNBC, Triple Negative Breast Cancer; TNM Plot, Tumor Node Metastasis Plot; UALCAN, The University Of Alabama At Birmingham Cancer Data Analysis Portal.
