# Supplementary figures and images for: Bioinformatics insights into TMPO-AS1–let-7b-5p–ESPL1/E2F8 regulatory axis in breast cancer
Source: Front Cell Dev Biol. 2025 Nov 5;13:1635862. doi: 10.3389/fcell.2025.1635862 (PMC12627056; doi:10.3389/fcell.2025.1635862)

## Slide 1
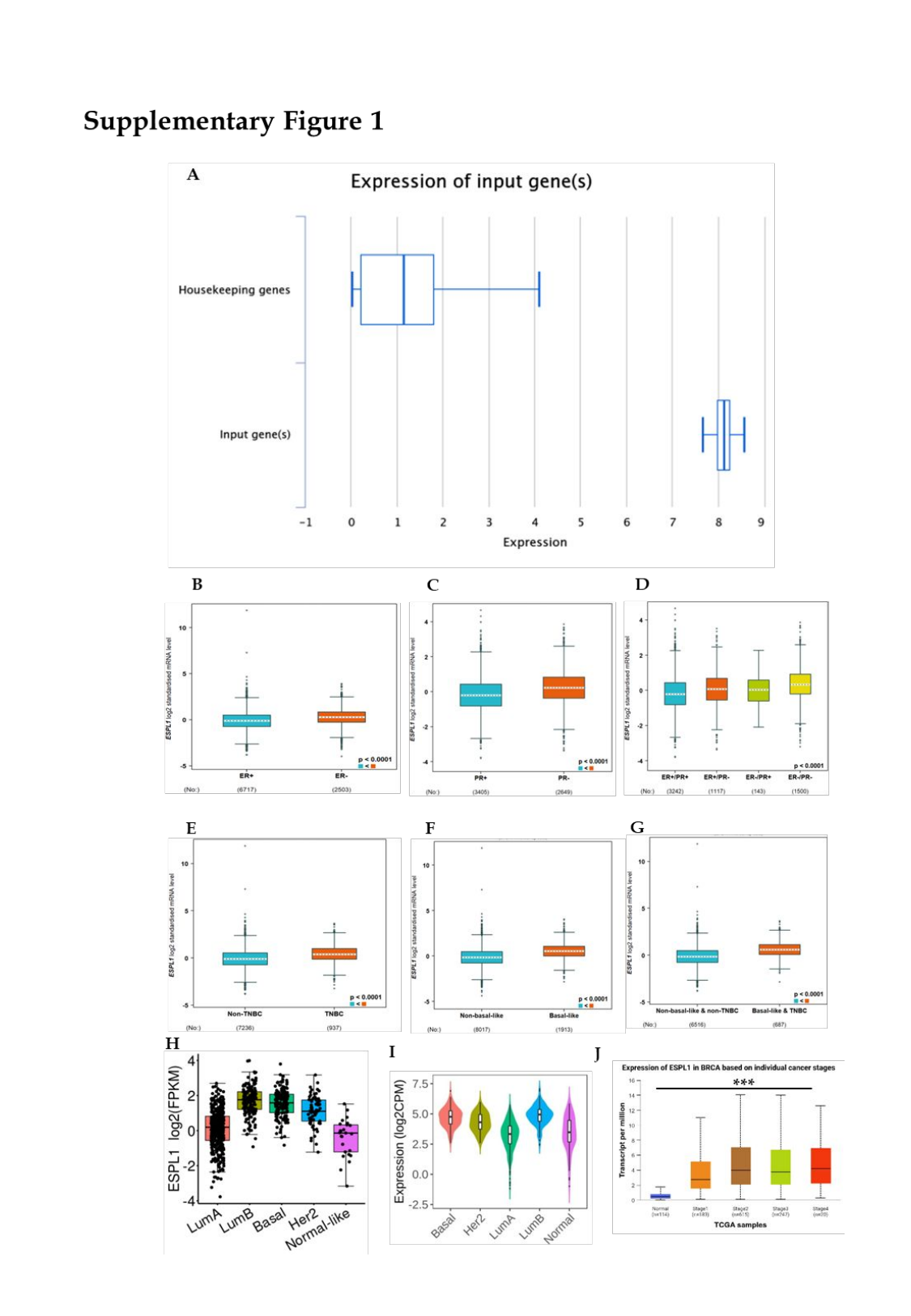

## Slide 2
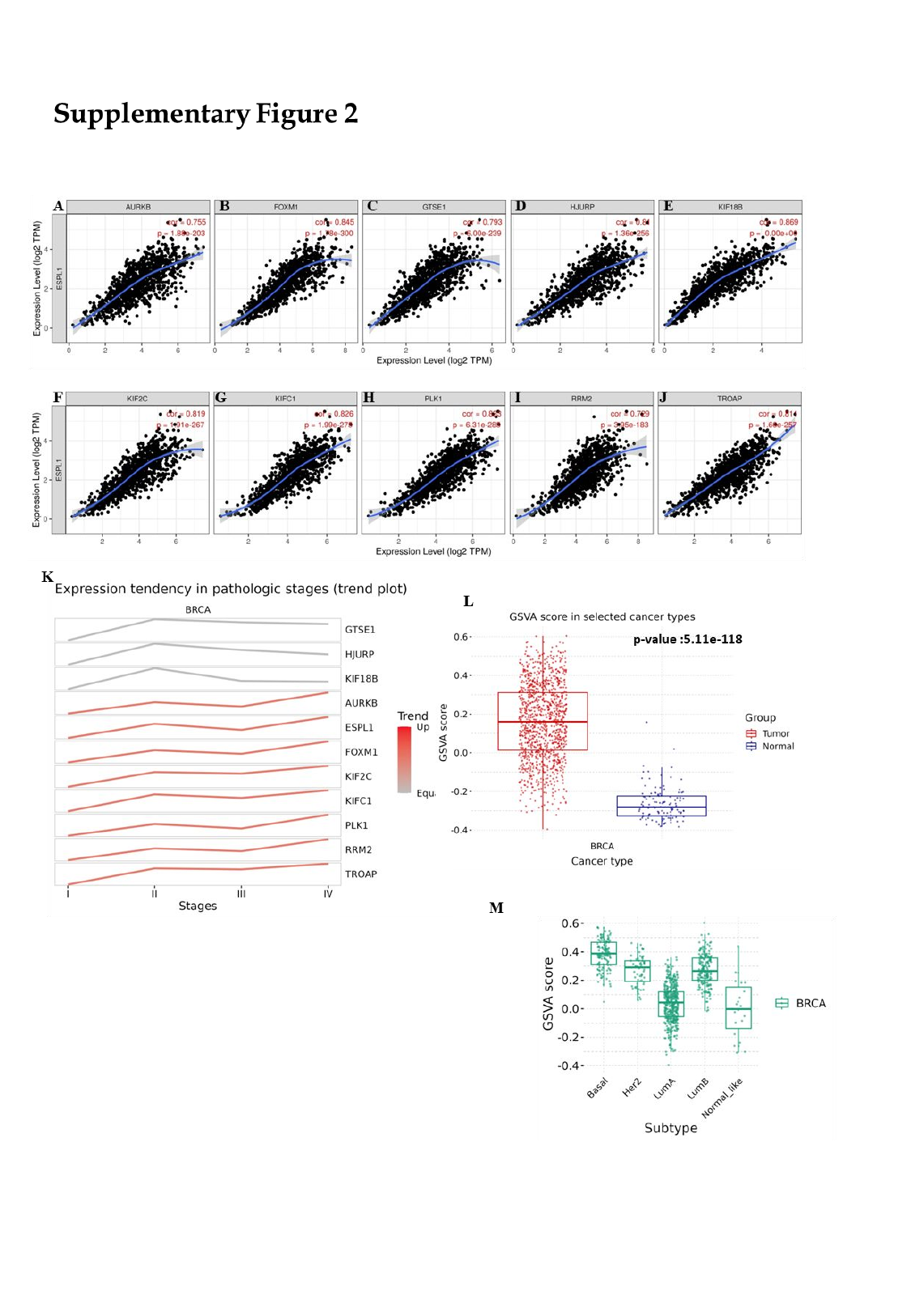

## Slide 3
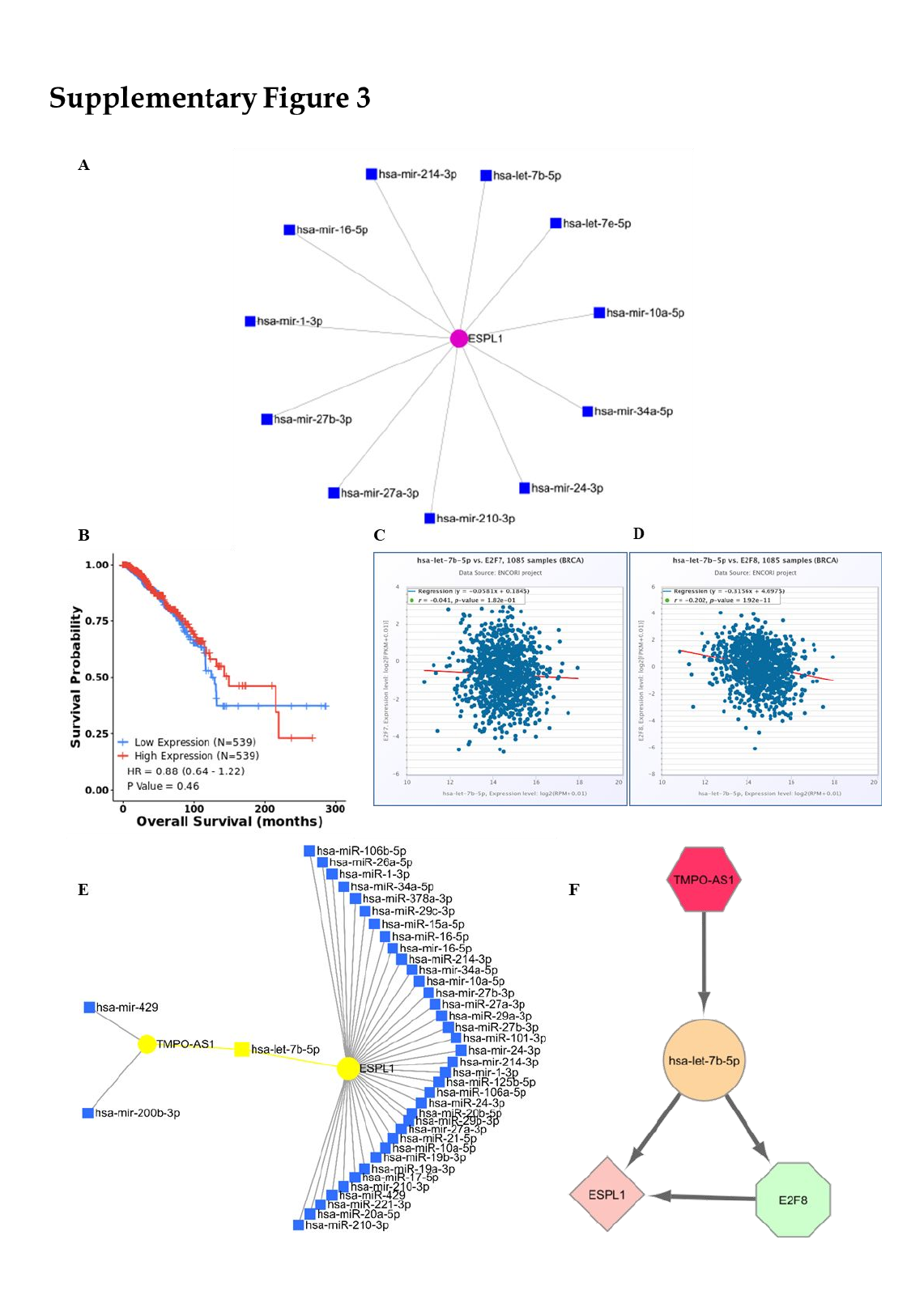

## Slide 4
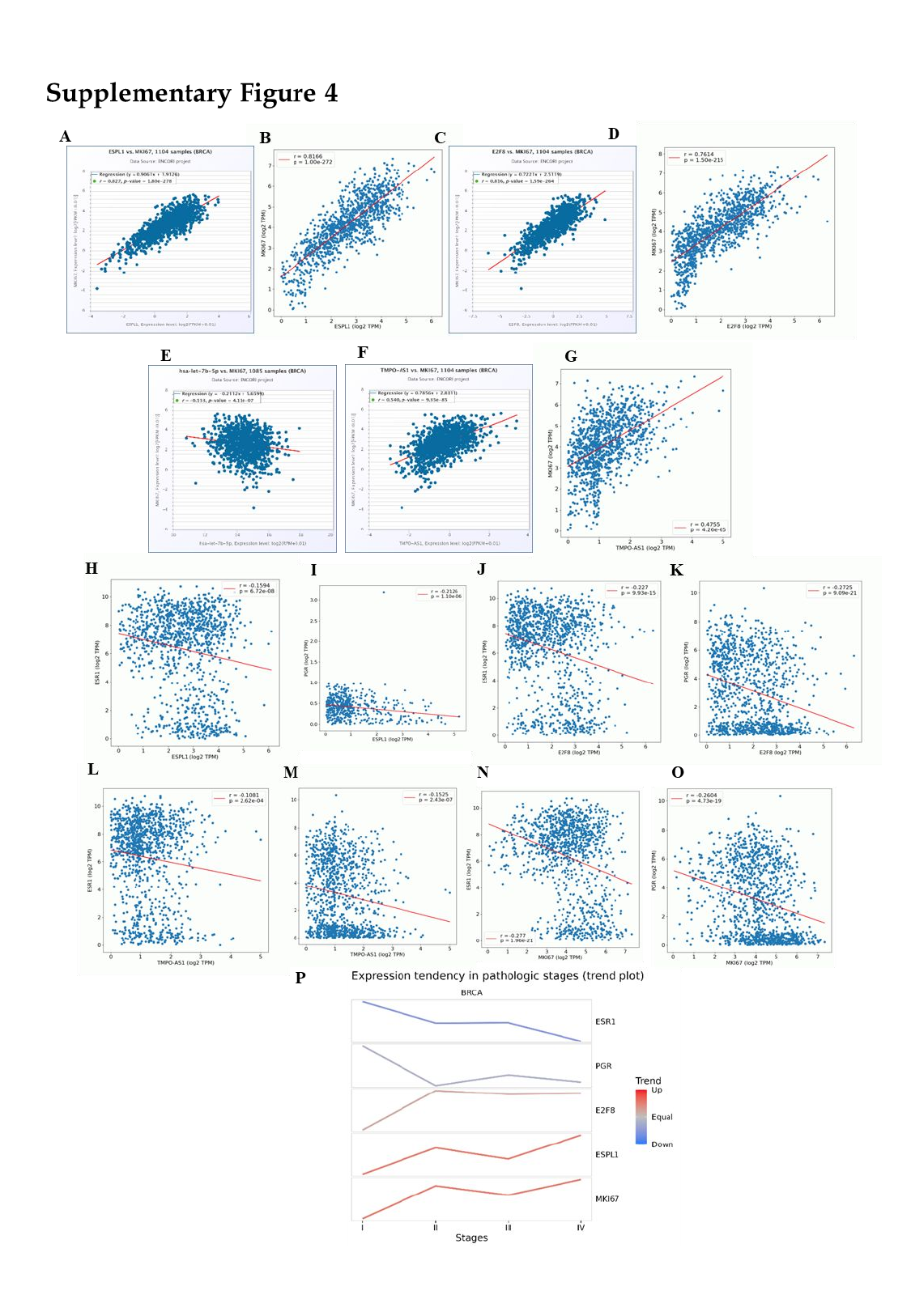

Supplement: Supplementary file 1 [file Presentation1.pptx]
