# Supplementary material for: Bioinformatics insights into TMPO-AS1–let-7b-5p–ESPL1/E2F8 regulatory axis in breast cancer
Source: Front Cell Dev Biol. 2025 Nov 5;13:1635862. doi: 10.3389/fcell.2025.1635862 (PMC12627056; doi:10.3389/fcell.2025.1635862)
Supplement: Supplementary file 2 [file DataSheet1.docx]

**Title:** **Bioinformatics Insights into TMPO-AS1–let-7b-5p–ESPL1/E2F8 Regulatory Axis in Breast Cancer**

**Authors:** Rajeev Nema^1#^, Prerna Vats^1#^, Aditi Singh^1^, Jaya Thilakan^2^, Swagata Brahmachari^3^, Pallavi Kulkarni^2^, Bhavika Baweja^1^, Chainsee Saini^1^, Sudhir K Goel^2^, Neha Arya^4^, Ashok Kumar^2^**^*^**

**Authors’ Affiliation:**

^1^Department of Biosciences Manipal University Jaipur, Dehmi Kalan, Jaipur-Ajmer Expressway, Jaipur, Rajasthan, 303007, India

^2^Department of Biochemistry, All India Institute of Medical Sciences (AIIMS), Bhopal, Saket Nagar, Bhopal 462 020, Madhya Pradesh, India

^3^Department of General Surgery, All India Institute of Medical Sciences (AIIMS), Bhopal, Saket Nagar, Bhopal 462 020, Madhya Pradesh, India

^4^Department of Translational Medicine, All India Institute of Medical Sciences (AIIMS), Bhopal, Saket Nagar, Bhopal 462 020, Madhya Pradesh, India

**#=**equivalent as first author

**Address for Correspondence:**

Dr. Ashok Kumar

Professor

Department of Biochemistry,

All India Institute of Medical Sciences (AIIMS) Bhopal,

Saket Nagar, Bhopal 462020, India

E-mail: [ashok.biochemistry@aiimsbhopal.edu.in](mailto:ashok.biochemistry@aiimsbhopal.edu.in)

**Supplementary Figure Legends**

**Supplementary Figure** 1. (A) Overexpression of ESPL1 as compared to the housekeeping genes by using CancerSEA. (B-G) mRNA expression of ESPL1 in BC aggressiveness and subtypes using bc-GenExMiner v5.0; (H-I) ESPL1 gene expression with the BC subtypes using the TCGA portal and TISIDB; (J) differential expression of ESPL1 in BC cancer stages using the UALCAN database.

**Supplementary Figure** 2. ESPL1’s co-expressed genes using (A-J) the TIMER 2.0 database, (K) GSCA database and (L-M) Co-expressed genes with normal vs. tumor and BRCA subtypes by the GSCA database.

**Supplementary Figure** 3: (A) miRNAs associated with the ESPL1 using the miRNet database (B) mir-10a-5p survival analysis using CancerMIRNome (C-D) Correlation of let-7b-5p with E2F7/E2F8 using the ENCORI database (E) The TMPO-AS1 lncRNA network was created with ESPL1/let-7b-5p using the miRNet database (F) TMPO-AS1/E2F8/ESPL1/hsa-let-7b-5p network created using Cytoscape.

**Supplementary Figure** 4. Molecular Mechanism of gene expression in BRCA. (A-B) ESPL1 vs MKI67, (C-D) E2F8 vs MKI67, (E) hsa-let-7b-5p vs MKI67, (F-G) TMPO-AS1 vs MKI67, Correlation with ESR1 and PGR, respectively (H-I) ESPL1, (J-K) E2F8, (L-M) TMPO-AS1, (N-O) MKI67. (P) Trend Plot for BC stages using GSCA.

**Supplementary Table 1: Stage and grade of the breast cancer patients**

| **S.No** | **Patient ID** | **TNM Staging** | **Grade of tumor** |
| --- | --- | --- | --- |
| 1. | B2 | T_3_N_1_M_0_ | II |
| 2. | B4 | T_2_N_1_M_0_ | I |
| 3. | B8 | T_3_N_1_M_0_ | II |
| 4. | B9 | T_2_N_1_M_0_ | II |

**Supplementary Table 2: ESPL1 Expression in TCGA Pan-Cancer**

| UniProtKB/SwissProt AC | Gene Symbol | Log2 F.C. | P-value | Adj. P-value | Significant | Expression Trend | TCGA Study | Source |
| --- | --- | --- | --- | --- | --- | --- | --- | --- |
| Q14674 | ESPL1 | 0.03 | 0.832 | 0.878 | No | up | thyroid cancer | TCGA |
|  |  | -0.24 | 0.241 | 0.328 | No | down | stomach cancer |  |
|  |  | 1.41 | 2.13E-11 | 1.60E-10 | Yes | up | liver cancer |  |
|  |  | 3.84 | 4.36E-97 | 6.71E-94 | Yes | up | uterine cancer |  |
|  |  | 1.58 | 1.53E-10 | 3.19E-09 | Yes | up | bladder cancer |  |
|  |  | -0.84 | 8.55E-09 | 5.18E-08 | Yes | down | head_and_neck  cancer |  |
|  |  | 3.45 | 6.35E-216 | 3.01E-213 | Yes | up | lung cancer |  |
|  |  | 1.64 | 1.68E-08 | 8.84E-07 | Yes | up | esophageal cancer |  |
|  |  | 1.1 | 3.87E-31 | 6.49E-30 | Yes | up | colorectal cancer |  |
|  |  | 1.26 | 3.14E-13 | 3.28E-12 | Yes | up | prostate cancer |  |
|  |  | -0.14 | 0.222 | 0.273 | No | down | kidney cancer |  |
|  |  | **2.72** | **1.68E-142** | **3.77E-140** | **Yes** | **up** | **breast cancer** |  |

**Supplementary Table 3: ESPL1 Survival Status**

| **S.No** | **Gene** | **Survival** | **Patients** | **Low Expression (Months)** | **High Expression (Months)** |
| --- | --- | --- | --- | --- | --- |
| 1. | ESPL1 | OS | 1879 | 118.62 | 70.62 |
| 2. |  | DMFS | 2756 | 130.72 | 51.88 |
| 3. |  | RFS | 4929 | 63 | 38.2 |

**Supplementary Table 4: ESPL1 Associated miRNAs**

| **S No.** | **Gene** | **miRNAs** | **R- Value** |
| --- | --- | --- | --- |
| 1. | ESPL1 | has-let-7b-5p | -0.267 |
| 2. |  | has-let-7e-5p | Positive |
| 3. |  | hsa-miR-10a-5p | -0.221 |
| 4. |  | hsa-miR-34a-5p | positive |
| 5. |  | hsa-miR-214-3p | -0.130 |
| 6. |  | hsa-miR-24-3p | positive |
| 7. |  | hsa-miR-210-3p | positive |
| 8. |  | hsa-miR-27a-3p | positive |
| 9. |  | hsa-miR-27b-3p | positive |
| 10 |  | hsa-miR-1-3p | -0.134 |
| 11. |  | hsa-miR-16-5p | positive |

**Supplementary Table 5: ESPL1 and Significantly Associated lncRNAs**

| **S.No** | **Gene** | **lncRNA** | **R-Value** |
| --- | --- | --- | --- |
| 1. | ESPL1 | DDX11-AS1 | 0.417 |
| 2. |  | TMPO-AS1 | 0.628 |
| 3. |  | DEPDC1-AS1 | 0.376 |
| 4. |  | LINC01775 | 0.478 |
| 5. |  | CSRP3 | 0.055 |

**Supplementary Table 6: lnRNA-TMPO-AS1 in Pan-Cancer Expression View**

| **Database ID** | **Symbol** | **Category** | **Species** | **Disease Name** | **Detection Method** | **Score** | **Causality** |
| --- | --- | --- | --- | --- | --- | --- | --- |
| LDA0006671 | TMPO-AS1 | LncRNA | Homo sapiens | Breast Neoplasms | Strong evidence | 0.999893 | Yes |
| LDA0006766 | TMPO-AS1 | LncRNA | Homo sapiens | Colorectal Neoplasms | Strong evidence | 0.999893 | Yes |
| LDA0006767 | TMPO-AS1 | LncRNA | Homo sapiens | Carcinoma, Hepatocellular | Strong evidence | 0.999893 | Yes |
| LDA0006874 | TMPO-AS1 | LncRNA | Homo sapiens | Urinary Bladder Neoplasms | Strong evidence | 0.999893 | Yes |
| LDA0006880 | TMPO-AS1 | LncRNA | Homo sapiens | Adenocarcinoma of Lung | Strong evidence | 0.999893 | Yes |
| LDA0006670 | TMPO-AS1 | LncRNA | Homo sapiens | Stomach Neoplasms | Strong evidence | 0.985791 | Yes |
| LDA0006673 | TMPO-AS1 | LncRNA | Homo sapiens | Esophageal Squamous Cell Carcinoma | Strong evidence | 0.985791 | Yes |
| LDA0006875 | TMPO-AS1 | LncRNA | Homo sapiens | Osteosarcoma | Strong evidence | 0.985791 | Yes |
| LDA0006672 | TMPO-AS1 | LncRNA | Homo sapiens | Carcinoma, Non-Small-Cell Lung | Strong evidence | 0.731059 | Yes |
| LDA0006872 | TMPO-AS1 | LncRNA | Homo sapiens | Glioma | Strong evidence | 0.731059 | Yes |
| LDA0006873 | TMPO-AS1 | LncRNA | Homo sapiens | Ovarian Neoplasms | Strong evidence | 0.731059 | Yes |
| LDA0006876 | TMPO-AS1 | LncRNA | Homo sapiens | Uterine Cervical Neoplasms | Strong evidence | 0.731059 | Yes |
| LDA0006877 | TMPO-AS1 | LncRNA | Homo sapiens | Nasopharyngeal carcinoma | Strong evidence | 0.731059 | Yes |
| LDA0006878 | TMPO-AS1 | LncRNA | Homo sapiens | gallbladder carcinoma | Strong evidence | 0.731059 | Yes |
| LDA0006879 | TMPO-AS1 | LncRNA | Homo sapiens | Thyroid Neoplasms | Strong evidence | 0.731059 | Yes |

**Supplementary Table 7: TMPO-AS1 and Corresponding Co-expressed genes Correlation**

| **Rank** | **Gene** | **Score** |
| --- | --- | --- |
| 1. | HASPIN | 0.6502 |
| 2. | KIF18B | 0.6333 |
| 3. | ESPL1 | 0.6305 |
| 4. | ARHGAP11A | 0.6226 |
| 5. | KIF11 | 0.6177 |
| 6. | SGO1 | 0.6128 |
| 7. | MKI67 | 0.6009 |
| 8. | NUSAP1 | 0.6304 |
| 9. | PLK1 | 0.59910 |
| 10. | CDT1 | 0.597 |

**
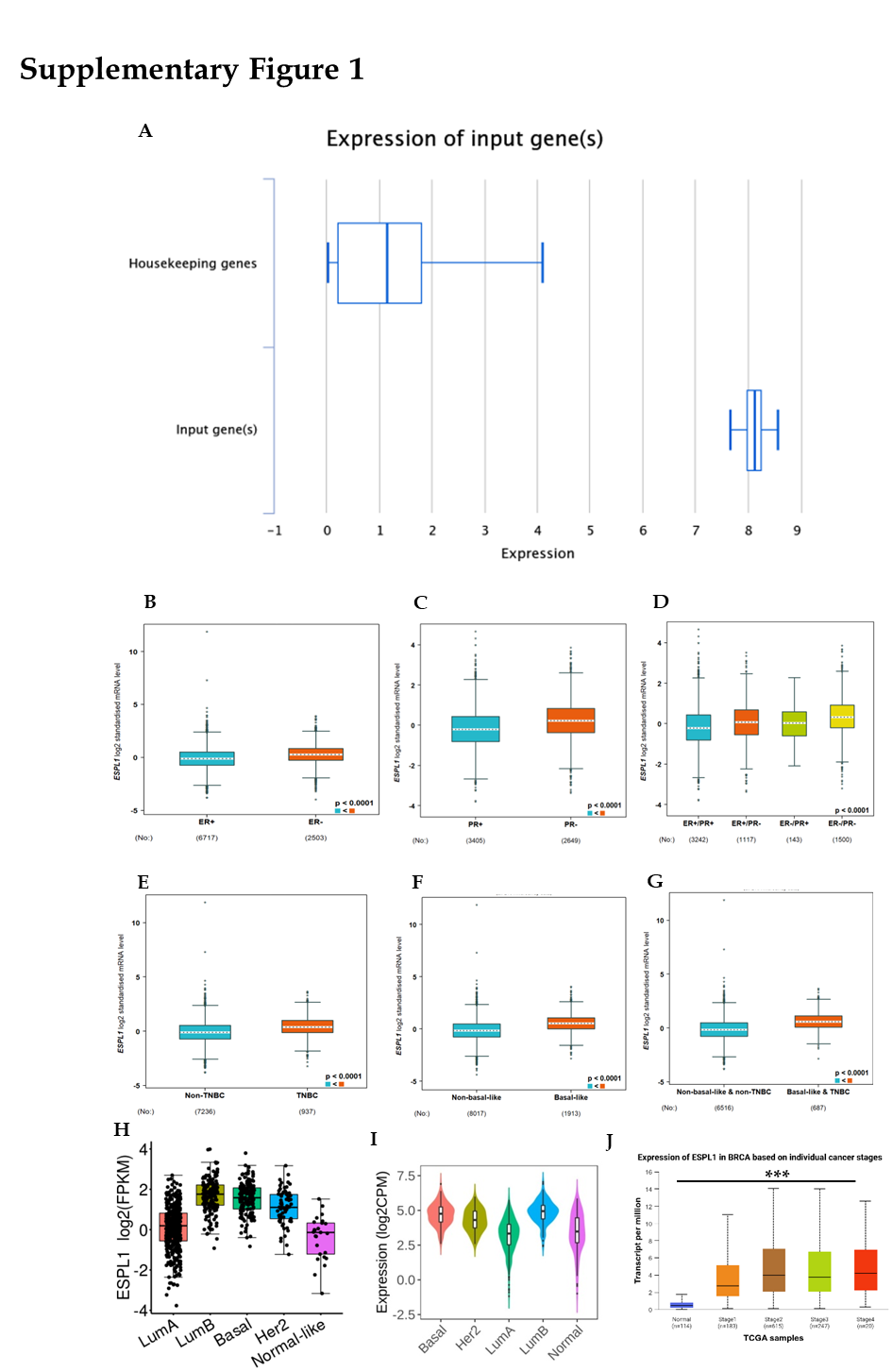
**

**
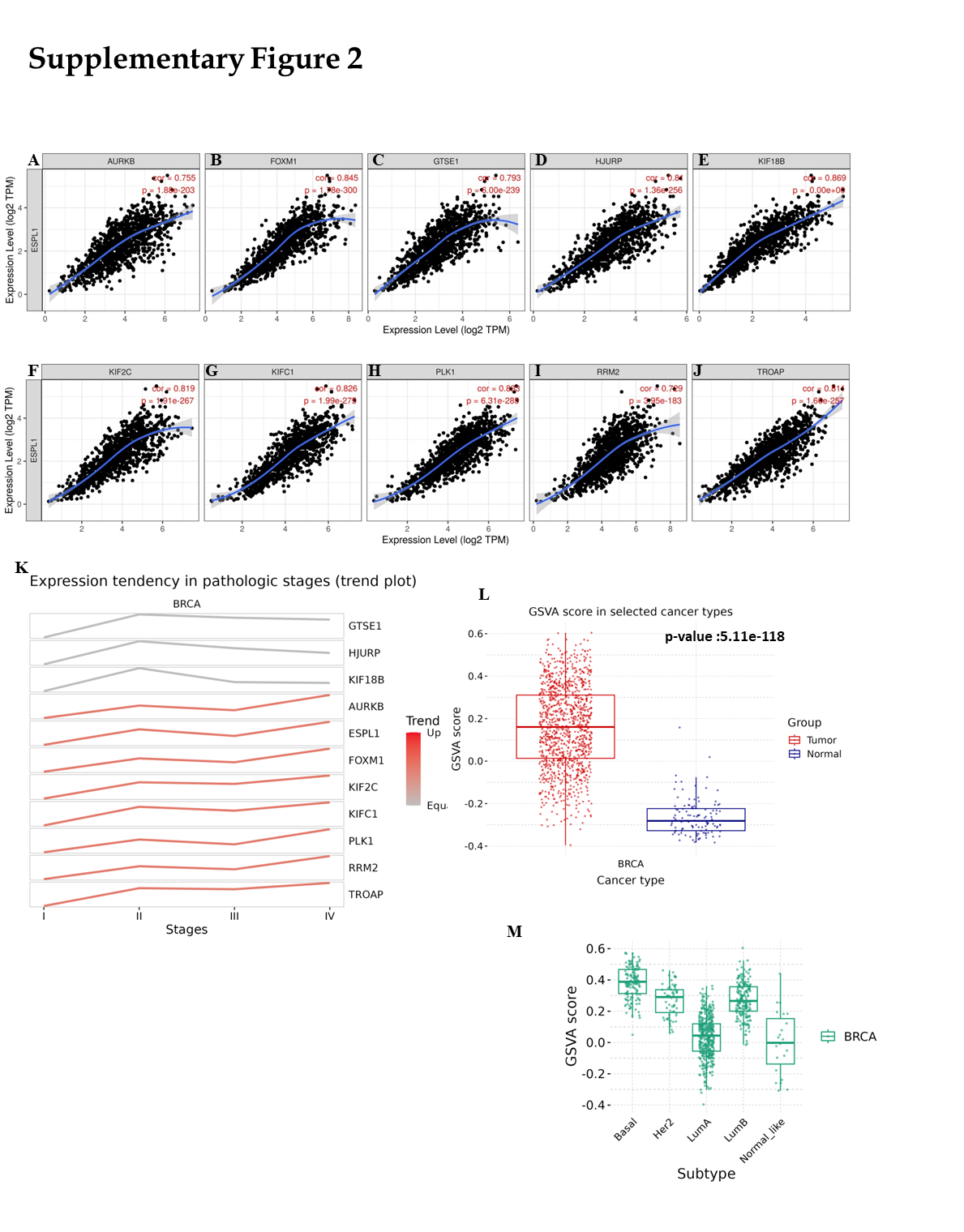
**

**
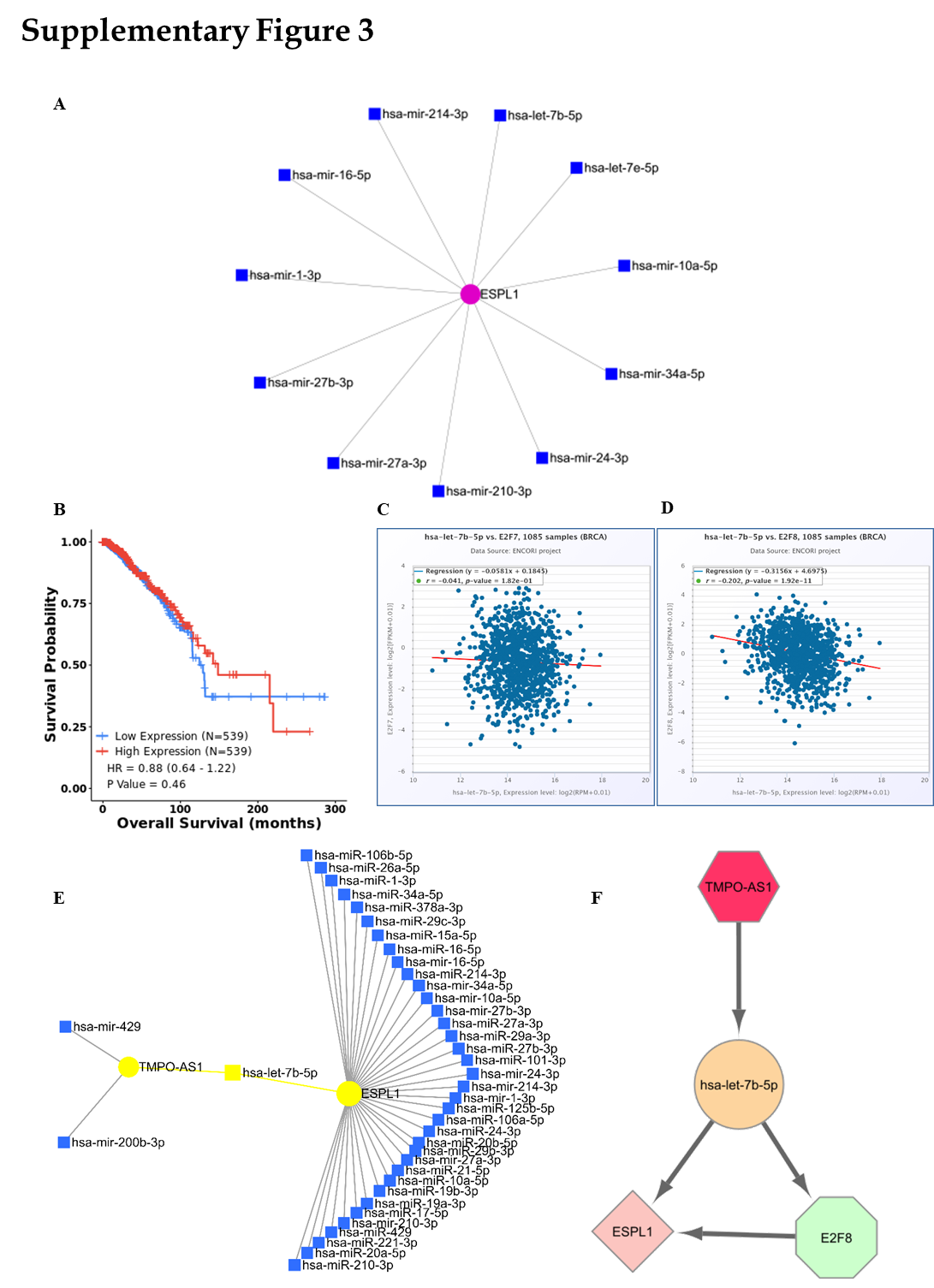
**

**
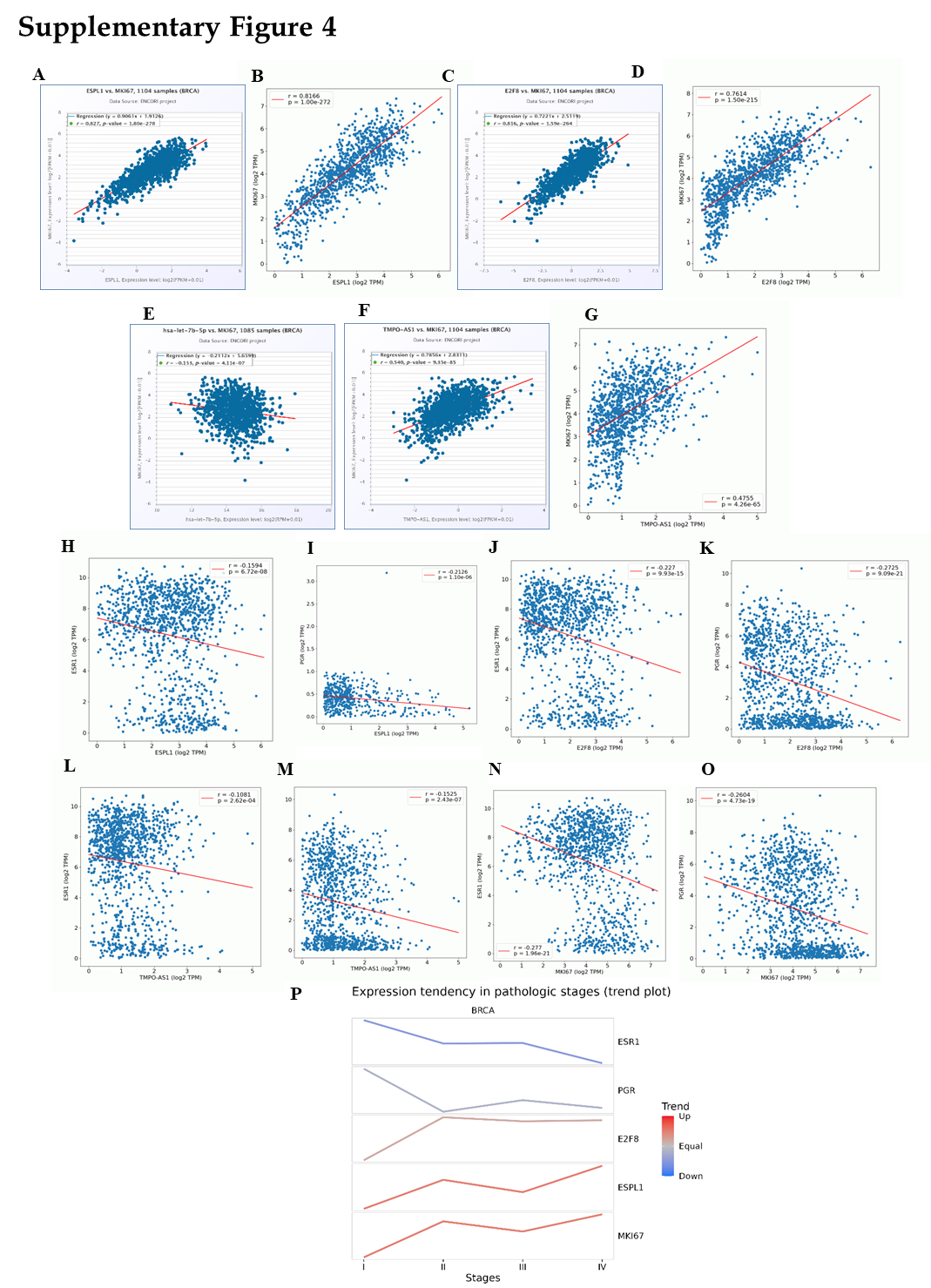
**
